# Computational exploration of molecular flexibility and interaction of meropenem analogs with the active site of oxacillinase-23 in *Acinetobacter baumannii*

**DOI:** 10.3389/fchem.2023.1090630

**Published:** 2023-02-23

**Authors:** Balajee Ramachandran, Saravanan Muthupandian, Jeyakanthan Jeyaraman, Bruno Silvester Lopes

**Affiliations:** ^1^ Structural Biology and Bio-Computing Lab, Department of Bioinformatics, Alagappa University, Karaikudi, Tamil Nadu, India; ^2^ Department of Pharmacology, Saveetha Institute of Medical and Technical Sciences (SIMATS), Chennai, Tamil Nadu, India; ^3^ School of Health and Life Sciences, Teesside University, Middlesbrough, United Kingdom; ^4^ National Horizons Centre, Teesside University, Darlington, United Kingdom

**Keywords:** *Acinetobacter baumannii*, beta-lactamases, oxacillinase, OXA-23, OXA-23 variants, meropenem, MM/GBSA

## Abstract

**Background:** Carbapenem-resistant *Acinetobacter baumannii* is an opportunistic pathogen responsible for nosocomial infections and is one of the biggest global threats according to the World Health Organization (WHO), particularly causing substantial morbidity and mortality.

**Objectives:** This study aimed at using computational approaches to screen meropenem and its analogs against OXA-23-positive *Acinetobacter baumannii,* analyzing the correlations between kinetic and phenotypic characteristics.

**Methods:** A total of 5,450 compounds were screened using virtual screening workflow (HTVS, Glide-SP, and Glide-XP) to identify the best compounds based on their binding energy and interactions against OXA-23 and OXA-27 as they had phenotypic data available. Molecular dynamics simulation and density functional theory (DFT) studies were performed from the outcome of molecular docking analysis.

**Results:** During simulations, meropenem and its analogs exhibited high-level stable interactions with Ser79, Ser126, Thr217, Trp219, and Arg259 of OXA-23. Meropenem displayed a CovDock energy of about −3.5 and −1.9 kcal mol^-1^ against OXA-23 and OXA-27, respectively. Among the 5,450 compounds, Pubchem_10645796, Pubchem_25224737, and ChEMBL_14 recorded CovDock energy between −6.0 and −9.0 kcal mol^-1^. Moreover, the infra-red (IR) spectrophotometric analysis revealed C=O and C-N atoms showing bands at 1,800 and 1,125 cm^-1^, respectively. These observed data are in congruence with the experimental observations.

**Conclusion:** The identified compounds showed good agreement with the spectrophotometric analysis using DFT methods. In the earlier studies, meropenem’s MIC value was 32 μg mL^−1^ in OXA-23-positive isolate A2265 compared to the MIC of 1 μg mL^−1^ in Δ*bla*_OXA-23_ A2265. Comparing the CovDock energy and hydrogen-bonding interactions, the predicted results are in good agreement with the experimental data reported earlier. Our results highlight the importance of OXA-23 molecular docking studies and their compliance with the phenotypic results. It will help further in developing newer antibiotics for treating severe infections associated with carbapenem-resistant *A. baumannii*.

## 1 Introduction

In the earlier days, due to the lack of awareness and advances in hospital settings, *Acinetobacter baumannii* was considered of little clinical importance. Nowadays, *A. baumannii* is considered as an opportunistic pathogen which shows high rates of mortality and morbidity, especially in immunocompromised individuals and patients with prolonged hospital stay (Kallen et al., 2010). In 2019, 1.2 million people died as a result of antimicrobial resistance, and *A. baumanni* was one of the six pathogens solely responsible for a quarter of a million deaths worldwide (Murray et al., 2022). *A. baumannii* causes a wide range of hospital and community-based infections which include pneumonia, urinary tract infections, bacteremia, meningitis, and skin/soft tissue infections (Gaynes et al., 2005). The growing resistance of *A. baumannii* to first-line antibiotics has produced a deadly combination of antimicrobial resistance and pathogenicity that plagues hospitals (Wu et al., 2016). The beta-lactam antibiotics such as penicillin, cephalosporins, and carbapenems which inactivate the D, D-transpeptidase enzyme, which is crucial in the cross linking of peptidoglycans and block polymerization resulting in cell death, are considered of little importance due to the repertoire of antimicrobial resistance genes in this bacterium (Irina Massova, 1998; Jan Walther-Rasmussen, 2006).

The enzymes which hydrolyze oxacillin and penicillin antibiotics are termed class D β-lactamases, otherwise called oxacillinases or OXA classes of enzymes. Some of the classes of these enzymes prevalent in *A. baumannii* include OXA-23, OXA-24/40, OXA-51, and OXA-58. The identified β-lactamases can also plasmid encode β-lactamases in Gram-negative bacteria and contain more variants under each group (Evans and Amyes, 2014). Each group has its unique properties of interacting with antibiotics; therefore, the mechanism of resistance to antibiotics is unique. Interestingly, the OXA-23 class comprises a heterogeneous group of enzymes where the corresponding genes from this class can be identified on chromosomes and plasmids. The group from the class D beta-lactamases is diversified in nature and can be differentiated by sequence identity (Malathi et al., 2016). Therefore, this group of enzymes is very sensitive when a single amino acid change occurs in the sequence which results in changes to their hydrolytic activities (Bush, 2013). The presence of deuterium in active drug molecules enhances potential benefits such as safety, efficacy, and tolerability of a therapeutic agent and decreases its toxic influence (Tung, 2010). Huang et al. (2021) reported that mass spectrometry analysis reveals that the flexibility of the interdomain region may be reduced upon snug binding to the class A β-lactamase TEM1 without any changes in SHV1 or PC1. However, the rigidity in the loop region 135–145 indicates the enhancement of inhibition of the β-lactamase inhibitory protein (BLIP) to class A beta-lactamases. Hence, the modification of drugs with deuterium has emerged as an essential requirement in medicinal chemistry research.

The identified compound (e.g., antibiotic) will target the OXA-23 and help in effectively overcoming resistance which can be investigated through biochemical, microbiological, and genetic studies (Bush et al., 1995). Prolonged usage of β-lactam antibiotics has resulted in the emergence of antibiotic-resistance bacterial strains. Carbapenem classes of antibiotics such as imipenem and meropenem are the drugs of choice for infections caused by multidrug-resistant bacteria (Jean-JacquesRouby et al., 2014; Sgrignani et al., 2016). Sgrignani et al. (2016)elucidated the hydrolytic mechanism of the OXA-23–meropenem complex and observed some critical issues such as (a) molecular variations that lack the recognition of β-lactams and (b) the modification of the structure of drugs that impact the identification of potential drugs. The role of some specific hydrogen bonds between antibiotics and other important residues such as Thr217 and Ser126 has also been emphasized (Pappa-Wallace et al., 2011). Hence, these interactions always influence the view of planning and structural modification to enhance their resistance to β-lactam-catalyzed hydrolysis. In *A. baumannii*, OXA-23 production causes imipenem and meropenem resistance with a minimum inhibitory concentration of >16 mg/L (Smith et al., 2013). The OXA-23 group is structurally related to other variants which include OXA-27, OXA-49, OXA-73, OXA-103, OXA-105, OXA-133, OXA-134, OXA-146, OXA-165, OXA-171, OXA-225, and OXA-239. The substitution of amino acids in OXA-23 leads to an understanding of structural features and functions. A quantum mechanical treatment was applied to meropenem and its analogs to investigate vibrations within the bonds using spectral methods. As a result of the spectrophotometric analysis, the C=O and N-H band was identified; it reveals the electrophilic and nucleophilic attack sites (Cielecka-Piontek et al., 2013).

**SCHEME 1:**
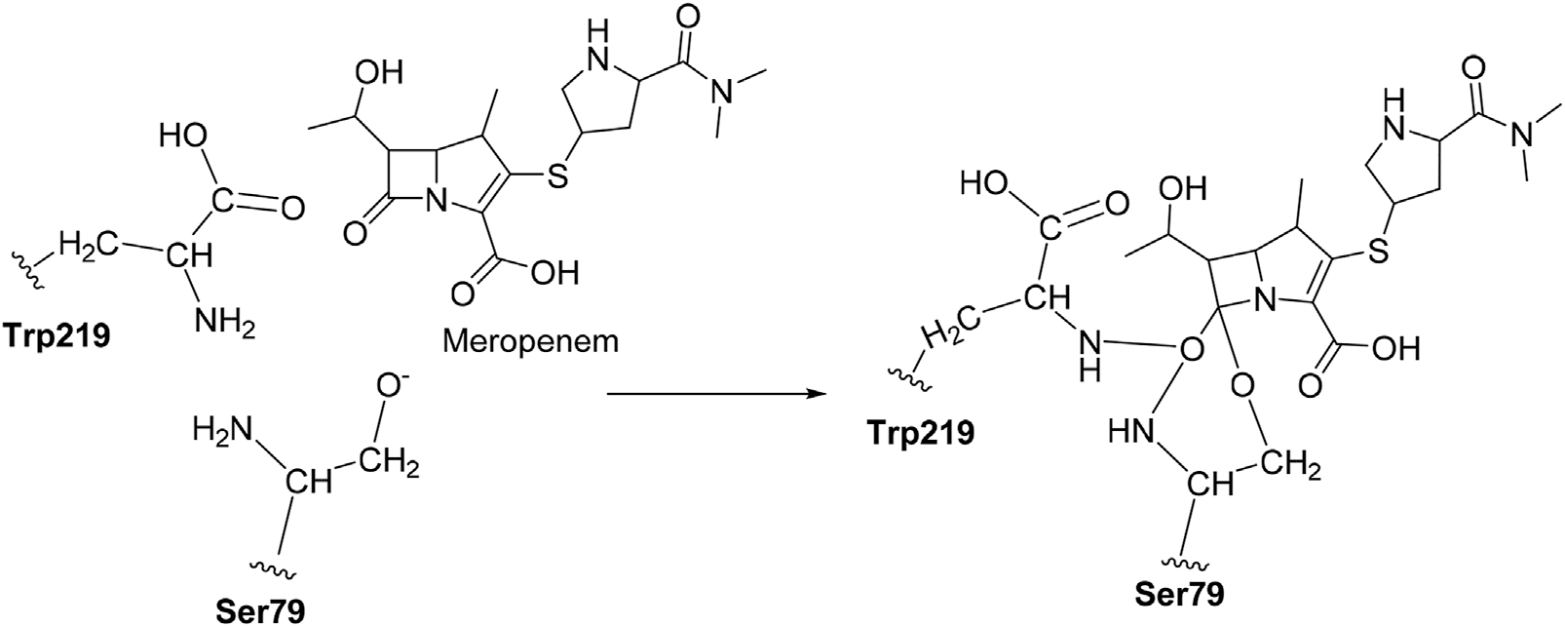
Mechanism for covalent binding between OXA-23 and meropenem.

This study aimed to screen the potential compounds active against *A. baumannii* using computational approaches, *viz.,* homology modeling, molecular docking, molecular dynamics simulation, MM/PBSA free energy calculations, and covalent docking to analyze the binding mechanism of OXA-23 and OXA-27 (Scheme 1). The quantum mechanical treatment is further employed to determine their electronic structural properties through HOMO-LUMO and the bands were analyzed through spectrophotometric analysis.

## 2 Materials and methods

### 2.1 Homology modeling of OXA-23 variants

The sequence of the variants was retrieved from UniProt (www.expasy.org), and sequence alignment was performed using BLASTP (www.ncbi.nlm.nih.gov/blastp). The multiple sequence alignment was performed to check their identical regions, and as a consequence, the secondary structure based on the alignment was predicted using the ESPript server (Xavier Robert, 2014). The availability of three-dimensional (3D) structures of the targeted proteins is a crucial step in the structure-based drug design. OXA-23 (PDB ID: 4JF4) was obtained from the Protein Data Bank (www.rcsb.org). The OXA-23 variants used in the study were those reviewed previously (Evans and Amyes, 2014). The structures of OXA-146 (4K0W), OXA-225 (4X55), and OXA-239 (5W1B) and the variants of OXA-23 were used from the Protein Data Bank (www.rcsb.org). The homology modeling approach using Swiss-Modeller was employed to construct the structure for OXA-27, OXA-49, OXA-73, OXA-103, OXA-133, OXA-134, OXA-165, OXA-171, and OXA-225 as these variants had no structures associated with them in the PDB. The modeled protein structure was validated by using Ramachandran plot analysis (http://services.mbi.ucla.edu/SAVES/Ramachandran/), ERRAT (http://services.mbi.ucla.edu/ERRAT/), and Verify3D (http://services.mbi.ucla.edu/Verify3D/).

### 2.2 Protein preparation step

The starting coordinates of OXA-23 and its variants (n = 12) were further analyzed for Glide docking calculations and subjected to protein preparation using the Protein Preparation Wizard of Schrodinger Suite (Schrodinger, LLC, New York, United States). Protein preparation is a prerequisite step to simulate accurate protein–ligand docking. (i) Water molecules are removed; (ii) hydrogen atoms are included; (iii) pH of the biological complex was maintained at 7.0 ± 2.0 using Epik; (iv) hydrogen atoms were added, and the orientation of hydroxyl groups in Asn and Gln and the protonation state of His were optimized to maximize hydrogen bonding; and (v) geometrical optimization was performed to a maximum RMSD of 0.3 Å with the OPLS-2005 (Optimized Potential for Liquid Simulation) force field (Guru Raj et al., 2016; Ramachandran et al., 2020).

### 2.3 Ligand preparation

The similar structures of meropenem were retrieved from the following online databases: (i) PubChem (www.ncbi.nlm.nih.gov/pubchem), (ii) ChEMBL (www.ebi.ac.uk/chembl/), (iii) ZINC (www.zinc.docking.org), and (iv) ChemBridge (www.chembridge.com) by applying a similarity cutoff of 60%. The obtained compounds are prepared using LigPrep v3.3, Schrodinger, LLC, New York, United States. The structures are processed with the following parameters: (i) OPLS-2005 force field, (ii) assigning a possible ionization state at pH 7.0 ± 2.0, and (c) tautomers were generated, and all the remaining parameters are kept as default (Sankar et al., 2022).

### 2.4 Protein grid generation

The active site of the protein contains important key residues such as Ser79, Lys82, Leu125, Ser126, Thr217, Trp219, and Arg259 and these sites were utilized to generate the grid box for the further use of molecular docking analysis (Zhu et al., 2014). The atoms of the proteins were scaled with van der Waal’s radius at a factor of 1 Å and a partial charge cutoff of 0.25 Å (Natarajan et al., 2020).

### 2.5 Virtual screening workflow

Virtual screening is the use of high-performance computing to analyze large datasets of chemical compounds to filter out the potential poses. The obtained compounds from the online sources are employed in the virtual screening workflow (Schrodinger LLC, New York, United States) against OXA-23. The screening was carried out step-by-step and the best docked poses are filtered using Glide score, Glide energy, and hydrogen bond interactions (Halgren et al., 2004; Friesner et al., 2006; Bochevarov et al., 2013; Balajee et al., 2016; Ramachandran et al., 2020).

### 2.6 Binding free energy calculations

The binding free energy calculation was performed to provide the energetic contributions of the receptor to the ligand binding of the docked complexes. The binding free energy was evaluated using molecular mechanics/Poisson–Boltzmann surface area (MM/PBSA) and molecular mechanics/generalized-Bonn surface area (MM/GBSA) methods which were performed using the MMPBSA.py module in Amber18. The energy breakdown for the per-residue basis was calculated based on electrostatic interactions, van der Waal’s energy, polar solvation energy, and non-polar solvation energy for residues with the binding energies.

### 2.7 Covalent docking

The CovDock module of Schrodinger, LLC, United States, was employed to perform covalent docking. The amino acid Ser79 was taken as a reactive residue which will have a nucleophilic interaction between the O^−^and carbonyl group of carbapenems as the electrophile. Here, we defined the reaction pattern using the Maestro format SMARTS pattern [C, c] = [O, S] as the nucleophilic addition step (Oyedele et al., 2022).

### 2.8 Analyzing the molecular docking data

We employed nAPOLI, a graph-based strategy to detect and visualize conserved protein–ligand interactions in large-scale (Fassio et al., 2020). It analyzes the protein–ligand interactions to understand the insights of the complex and has an important feature which is used to detect the type of hydrophobic interactions and hydrogen bonding with the specific atom.

### 2.9 Molecular dynamics

The MD simulations were carried out using Desmond simulation package of Schrodinger LLC, NY, United States. The constant-temperature and constant-pressure ensemble (NPT) allows control over both the temperature and pressure when a temperature of 300 K and a pressure of 1 bar were applied in all the runs. The simulation length was 100 ns for the apoprotein and 50 ns for the protein–ligand complex with a relaxation time of 1 ps. The OPLS-2005 force field parameters were used in all simulations. The system was set up for simulation using a predefined water model (simple point charge, SPC) as a solvent in the orthorhombic box with periodic boundary conditions and the overall charge of the system was neutralized by adding Na^+^ ions. The relaxation of the system was achieved by implementing the steepest descent algorithms. The long-range electrostatic interactions were calculated using the particle mesh Ewald method. The cut-off radius in Coulomb interactions was 9.0 Å (Ramachandran et al., 2022a). The water molecules were explicitly described using the simple point charge model. The Martyna−Tuckerman−Klein chain coupling scheme with a coupling constant of 2.0 ps was used for the pressure control and the Nosé−Hoover chain coupling scheme was used for the temperature control (Martyna and Klien, 1992). Non-bonded forces were calculated using an r-RESPA integrator where the short range forces were updated every step and the long-range forces were updated every three steps. The trajectories were saved at 4.8-ps intervals for analysis. The behaviour and interactions between the ligands and protein were analyzed using the Simulation Interaction Diagram tool implemented in the Desmond MD package (Ramachandran et al., 2022b). The stability of MD simulations was monitored by observing the root mean square deviation (RMSD) of the ligand and protein atom positions at the specific time.

### 2.10 Density functional theory (DFT) calculations

The DFT calculations were employed by using Jaguar, version 8.7 (Schrödinger, LLC, New York, NY) (Bochevarov et al., 2013). Initial geometry was optimized using B3LYP/6-31+G* methods and basis sets (Cielecka-Piontek, et al., 2013; Abdullah et al., 2022). The compounds which showed better binding affinity in Glide-XP were taken as the input for the DFT calculations. This method elaborates the behaviour of ligands from electron density, HOMO-LUMO, and MESP (Balajee et al., 2016). Furthermore, the experimental values reported by Cielecka-Piontek et al. (2013) were compared with the theoretical values. Meropenem and the selected analogs Pubchem_67943222, ChEMBL_14, Pubchem_10645796, and Pubchem_25224737 were interpreted for the IR absorption.

## 3 Results and discussion

### 3.1 Sequence alignment and model generation

The structure of OXA-23 variants was generated based on the OXA-23 structure (PDB ID: 4JF4). Sequence alignment plays a vital role in selecting the template for homology modeling (Supplementary Figure S1). The similarity between the template and target sequences was observed to be more than 30% using BLASTP search and validated. The alignment of OXA-23 variants reveals that the overall structural fold was similar and the active site residues were highly conserved.

### 3.2 Validation of modeled structures

The ERRAT online server demonstrates that the modeled protein structures were stabilized by local interactions and the overall quality factor was 91.200 for the selected protein structure which is acceptable (Supplementary Figure S2A). The Verify3D tool evaluates the protein structure by utilizing three-dimensional profiles. This program examines by comparing the 3D–1D score. The score ranges from −1 (poor score) to +1 (good score), where 98% of the sequence was found in the middle value of 3D–1D score >=0.2 that is perceptive for our demonstrated protein shown in Supplementary Figure S2B. Then, Ramachandran plot analysis using PROCHECK was employed to validate the structure. An assessment shows more than 93% of the residues fall in the favored region, with 5% in the allowed regions, while only few residues appeared in other regions (Supplementary Figure S2C), Furthermore, the modeled structure was superimposed with OXA-23 and the RMSD which resulted in 0.3 Å shown in Supplementary Figure S2D. Evans and Amyes (2014) [7] highlighted that the K_m_ values of meropenem with OXA-23 and OXA-27 were about 1 and 15 (µM), respectively. Hence, we initiated an investigation with OXA-27 and applied the same criteria as performed for OXA-23. Also, the variants of the OXA-23 have not been studied elsewhere using a computational approach to the best of our knowledge.

### 3.3 Reproducing the crystal structure (OXA-23)

In order to elucidate the mechanism of OXA-23 with meropenem and its analogs, the virtual screening protocol (Glide-HTVS, SP, and XP) was carried out and the docking protocol was initiated into the active site pocket as reported for OXA-23. The residues Ser79, Lys82, Arg259, Leu125, Lys124, Thr217, Ser126, and Trp219 interacted with meropenem and were taken as the active site residues.

Initially, the bond between the meropenem and Ser79 was cleaved from the crystal structure of OXA-23 (PDB ID: 4JF4) and it was minimized. The distance between Ser79 O^−^ and carbonyl carbon was about 3.2 Å and was observed after the minimization of the non-covalent complex. Then, meropenem was separated from the OXA-23 complex and it was re-docked which resulted in the distance of 3.0 Å. We have searched for the analogs (reference as meropenem) from the online database with a threshold cutoff of 60%.

#### 3.3.1 Molecular docking analysis of OXA-23

The compounds Pubchem_67943222, Pubchem_25224737, Pubchem_10645796, ChEMBL_14, Zinc_11, and Zinc_18 have shown consistent interactions with Arg259, Leu125, Lys124, Thr217, Ser126, Trp219, and Ser79. The Glide Score ranging from −9.0 to −5.0 kcal mol^-1^ and the Glide Energy ranging from −45 kcal mol^-1^ and -50 kcal mol^-1^ are shown in Table 1. The non-covalent interaction between the catalytic Ser79 O- and carbonyl group shows a distance of about 3.0 Å. This analysis provided a basic understanding of the significance of OXA-27 with the aforementioned compounds.

**TABLE 1:** GLIDE (HTVS, SP, and XP) outcome of the screened compounds.

Sl. no.	Protein	Compound	Docking score	Glide score	Glide energy
1	**OXA-23**	Meropenem	−7.600	−7.630	−50.733
2		Pubchem_67943222	−8.894	−8.894	−41.598
3		Pubchem_25224737	−8.742	−9.172	−47.578
4		Pubchem_10645796	−8.712	−9.121	−47.109
5		ChEMBL_14	−5.622	−5.622	−45.061
1	**OXA-27**	Meropenem	−5.571	−5.600	−39.640
2		Pubchem_67943222	−6.946	−6.946	−46.602
3		Pubchem_25224737	−6.776	−7.176	−49.597
4		Pubchem_10645796	−6.483	−6.883	−48.802
5		ChEMBL_14	−9.128	−9.177	−52.535

#### 3.3.2 Molecular docking analysis of OXA-27

The aforementioned compounds, meropenem, Pubchem_67943222, Pubchem_25224737, Pubchem_10645796, ChEMBL_14, Zinc_11, and Zinc_18 were docked into the active site of OXA-27. The Glide Score ranging from −9.0 to −5.0 kcal mol^-1^, docking score ranging from −8.0 and −6.0 kcal mol^-1^, and the Glide Energy ranging from −39 kcal mol^-1^ to −52 kcal mol^-1^ are shown in Table 1. By considering the Glide Energy values, the compounds Pubchem_67943222, Pubchem_25224737, Pubchem_10645796, and ChEMBL_14 were taken for further analysis. The screened compounds along with the chemical compound name and SMILE notation are given in Supplementary Table S1.

### 3.4 Per-residue energy calculation

**FIGURE 1:**
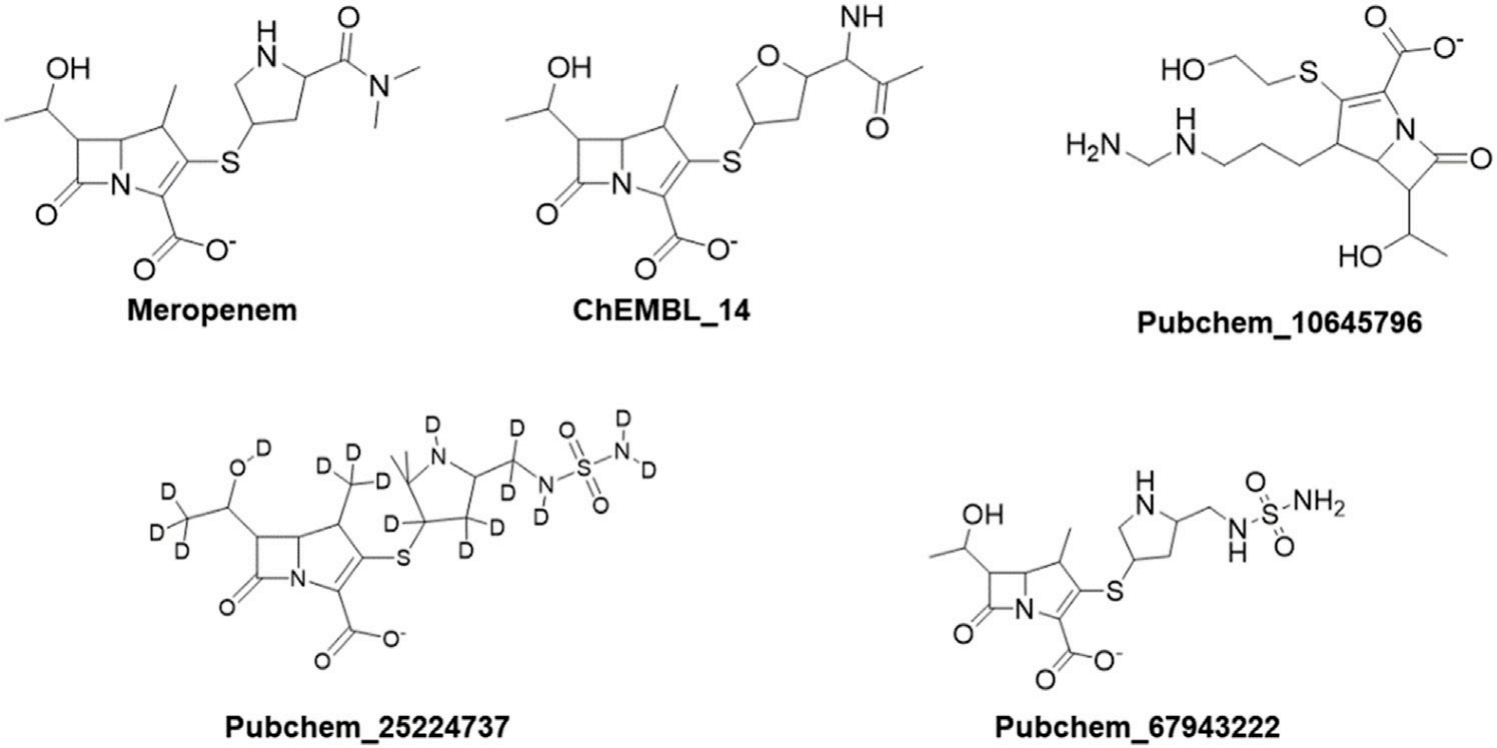
Resulting compounds from Glide-XP screening taken for covalent docking analysis.

The per-residue energy decomposition (PRED) was computed by MM/PBSA after 50-ns MD simulations of the complex (OXA-23/27 with meropenem and analogs). We have collected the common residual free energy information to understand their contribution in the simulation. The ΔG_bind_ of OXA-23 meropenem and OXA-27 meropenem is −16.22 kcal/mol and −17.57 kcal/mol, respectively. The compounds such as ChEMBL_14, Pubchem_25224737, Pubchem_10645796, and Pubchem_67943222 showed −34.80 kcal/mol, −28.79 kcal/mol, −25.72 kcal/mol, and −19.94 kcal/mol, respectively, against OXA-27. Furthermore, the decomposition analysis reveals that the catalytic serine (Ser-79) showed a value of −1.98 kcal/mol for OXA-23 and -1.20 kcal/mol for OXA-27. ChEMBL_14, Pubchem_10645796, Pubchem_25224737, and Pubchem_67943222 showed −4.24 kcal/mol, −3.52 kcal/mol, −3.34 kcal/mol, and −2.94 kcal/mol, respectively. Apart from the catalytic serine (Ser-79), the other residues such as Ser126, Thr217, Trp219 and Arg259 showed the highest energy and play a crucial role in terms of stabilizing the complexes. The interacting residues and their detailed energy contribution analysis are given in Table 2. Hence, the compounds such as meropenem, Pubchem_67943222, Pubchem_25224737, Pubchem_10645796, and ChEMBL_14 are taken to perform CovDock (covalent docking) analysis (Figure 1). This examination is to understand how meropenem and its analogs are stabilized in the pocket. The energy value was identified for the covalently bound complex through the CovDock protocol.

**TABLE 2:** Binding free energy decomposition of OXA-23 and OXA-27 with meropenem and its analogs.

OXA-23_meropenem					
**Residue**	**vdW**	**Electrostatic**	**Polar**	**Non-polar**	**Total**
Ser79	1.13 ± 0.06	−2.60 ± 0.12	1.82 ± 0.08	−0.07 ± 0.002	−1.98 ± 0.06
Thr80	0.11 ± 0.002	−0.13 ± 0.015	0.40 ± 0.018	0.0 ± 0.0	0.15 ± 0.007
Phe81	0.034 ± 0.000	−0.32 ± 0.007	0.354 ± 0.009	0.0 ± 0.0	−0.003 ± 0.00
Lys82	0.38 ± 0.011	0.12 ± 0.03	−0.07 ± 0.02	−0.003 ± 0.00	−0.33 ± 0.03
Lys124	0.17 ± 0.014	−0.22 ± 0.09	0.47 ± 0.08	−0.02 ± 0.003	0.06 ± 0.013
Leu125	1.01 ± 0.06	−2.23 ± 0.16	1.8 ± 0.07	−0.15 ± 0.01	−1.6 ± 0.17
Ser126	2.0 ± 0.05	0.64 ± 0.08	0.22 ± 0.07	−0.19 ± 0.00	−1.32 ± 0.06
Thr217	0.99 ± 0.03	0.16 ± 0.19	−0.18 ± 0.16	0.00 ± 0.00	−1.01 ± 0.06
Trp219	2.72 ± 0.11	−3.0 ± 0.2	3.3 ± 0.09	−0.29 ± 0.005	−2.8 ± 0.1
Arg259	0.15 ± 0.09	−14.8 ± 0.22	14.3 ± 0.22	−0.08 ± 0.002	−0.70 ± 0.11

OXA-27_meropenem					
**Residue**	**vdW**	**Electrostatic**	**Polar**	**Non-polar**	**Total**
Ser79	1.10 ± 0.06	−2.01 ± 0.17	1.97 ± 0.14	−0.07 ± 0.002	−1.20 ± 0.090
Thr80	0.10 ± 0.002	−0.11 ± 0.002	0.27 ± 0.020	0.01 ± 0.00	0.07 ± 0.004
Phe81	0.03 ± 0.00	−0.37 ± 0.01	0.39 ± 0.01	0.0 ± 0.0	−007 ± 0.001
Lys82	0.5 ± 0.02	−2.18 ± 0.08	1.54 ± 0.04	−0.01 ± 0.001	−1.13 ± 0.05
Lys124	0.09 ± 0.006	−2.01 ± 0.11	2.16 ± 0.11	−0.008 ± 0.002	0.04 ± 0.009
Leu125	0.28 ± 0.017	−0.18 ± 0.05	0.28 ± 0.045	−0.018 ± 0.002	−019 ± 0.24
Ser126	0.97 ± 0.08	−3.81 ± 0.40	2.5 ± 0.17	−0.17 ± 0.003	−2.44 ± 0.21
Thr217	0.41 ± 0.08	−6.62 ± 0.25	1.39 ± 0.15	−0.06 ± 0.005	−5.7 ± 0.154
Trp219	3.78 ± 0.108	−4.48 ± 0.14	3.5 ± 0.10	−0.36 ± 005	−5.11 ± 0.101
Arg259	0.42 ± 0.09	−2.5 ± 0.44	18.6 ± 0.23	−0.09 ± 0.003	−6.0 ± 0.2

OXA-27_ChEMBL14					
**Residue**	**vdW**	**Electrostatic**	**Polar**	**Non-polar**	**Total**
Ser79	−1.33 ± 0.071	−8.05 ± 0.32	5.23 ± 0.15	−0.083 ± 0.002	−4.24 ± 0.200
Thr80	0.11 ± 0.001	−1.6 ± 0.03	1.84 ± 0.03	0.0 ± 0.14	0.04 ± 0.006
Phe81	0.04 ± 0.0004	0.97 ± 0.015	0.96 ± 0.013	0.0 ± 0.0	−0.07 ± 004
Lys82	−0.4 ± 0.035	−4.16 ± 0.08	2.63 ± 0.03	−0.013 ± 0.00	−1.93 ± 0.06
Lys124	−0.11 ± 0.005	−13.36 ± 0.152	13.48 ± 0.152	−0.003 ± 0.001	0.007 ± 0.007
Leu125	−0.71 ± 0.028	−0.16 ± 0.58	0.29 ± 0.06	−0.103 ± 0.004	−0.68 ± 0.03
Ser126	−0.28 ± 0.15	−8.1 ± 0.19	3.8 ± 0.07	−0.12 ± 0.004	−4.7 ± 0.101
Thr217	−0.52 ± 0.08	−7.75 ± 0.23	2.11 ± 0.15	−0.21 ± 0.00	−6.18 ± 0.13
Trp219	−2.26 ± 0.09	−1.27 ± 1.69	1.69 ± 0.11	−0.25 ± 0.007	−2.085 ± 0.07
Arg259	0.48 ± 0.10	−46.8 ± 0.3	39.4 ± 0.18	−0.09 ± 0.03	−7.04 ± 0.19

**TABLE 2:** (*Continued*) Binding free energy decomposition of OXA-23 and OXA-27 with meropenem and its analogs.

OXA-27–Pubchem_25224737					
**Residue**	**vdW**	**Electrostatic**	**Polar**	**Non-polar**	**Total**
Ser79	−2.40 ± 0.54	−2.67 ± 0.10	1.8 ± 0.06	−0.07 ± 0.0	−3.33 ± 0.06
Thr80	−0.13 ± 0.002	−0.14 ± 0.025	0.48 ± 0.03	0.0 ± 0.0	0.21 ± 0.011
Phe81	−0.04 ± 0.00	−0.55 ± 0.012	0.57 ± 0.01	0.0 ± 0.00	0.02 ± 0.004
Lys82	−0.40 ± 0.01	0.18 ± 0.06	0.04 ± 0.04	0.0 ± 0.0	−0.19 ± 0.04
Lys124	−0.4 ± 0.02	−0.7 ± 0.10	1.53 ± 0.10	−0.14 ± 0.00	0.31 ± 0.03
Leu125	−0.5 ± 0.03	−1.17 ± 0.06	−1.2 ± 0.06	−0.05 ± 0.004	−0.5 ± 0.03
Ser126	−0.3 ± 0.12	−8.0 ± 0.15	3.43 ± 0.06	−0.2 ± 0.003	−4.75 ± 0.08
Thr217	−1.03 ± 0.101	−9.6 ± 0.19	1.62 ± 0.09	−0.14 ± 0.002	−9.13 ± 0.15
Trp219	−2.3 ± 0.08	−2.0 ± 0.11	2.4 ± 0.08	−0.24 ± 0.006	−2.12 ± 0.06
Arg259	0.53 ± 0.11	−25.7 ± 0.3	16.35 ± 0.2	−0.14 ± 0.004	−9.0 ± 0.2

OXA-27—Pubchem_67943222					
**Residue**	**vdW**	**Electrostatic**	**Polar**	**Non-polar**	**Total**
Ser79	−2.44 ± 0.05	−2.46 ± 0.12	2.1 ± 0.09	−0.14 ± 0.003	−2.94 ± 0.08
Thr80	−0.9 ± 0.002	−0.6 ± 0.15	0.8 ± 0.02	0.0 ± 0.0	0.1 ± 0.006
Phe81	−0.03 ± 0.00	−0.34 ± 0.006	0.35 ± 0.007	0.0 ± 0.0	0.03 ± 0.00
Lys82	0.2 ± 0.00	−0.41 ± 0.03	0.56 ± 0.03	0.0 ± 0.0	−0.025 ± 0.00
Lys124	−0.02 ± 0.001	−1.12 ± 0.034	1.20 ± 0.03	0.0 ± 0.0	0.02 ± 0.001
Leu125	−0.12 ± 0.01	0.07 ± 0.05	0.056 ± 0.05	−0.01 ± 0.025	−0.005 ± 0.00
Ser126	−0.53 ± 0.18	−1.1 ± 0.4	1.47 ± 0.45	−0.09 ± 0.02	−0.24 ± 0.17
Thr217	−0.84 ± 0.2	0.7 ± 1.2	−0.5 ± 1.0	−0.05 ± 0.02	−0.7 ± 0.3
Trp219	−2.7 ± 0.5	−3.3 ± 0.65	4.0 ± 0.7	−0.3 ± 0.003	−2.2 ± 0.4
Arg259	−0.07 ± 0.6	−14 ± 0.4	12.9 ± 0.28	−0.11 ± 0.001	−1.3 ± 0.1

OXA-27–Pubchem_10645796					
**Residue**	**vdW**	**Electrostatic**	**Polar**	**Non-polar**	**Total**
Ser79	−1.25 ± 0.08	−5.5 ± 0.30	3.69 ± 0.17	−0.07 ± 0.003	−3.52 ± 0.20
Thr80	−0.10 ± 0.00	−0.99 ± 0.02	1.32 ± 0.03	0.0 ± 0.0	0.23 ± 0.01
Phe81	−0.04 ± 0.00	−0.6 ± 0.01	0.6 ± 0.07	0.0 ± 0.0	−0.05 ± 0.004
Lys82	−0.42 ± 0.03	−2.5 ± 0.09	1.43 ± 0.04	−0.01 ± 0.00	−1.52 ± 0.07
Lys124	−0.14 ± 0.01	−0.04 ± 0.07	0.27 ± 0.07	−0.02 ± 0.0003	0.07 ± 0.007
Leu125	−0.96 ± 0.08	−1.6 ± 0.08	2.1 ± 0.1	−0.13 ± 0.009	−0.61 ± 0.05
Ser126	−0.18 ± 0.12	−8.7 ± 0.2	4.13 ± 0.10	−0.14 ± 0.003	−4.9 ± 0.1
Thr217	−0.45 ± 0.08	−8.0 ± 0.25	1.8 ± 0.15	−0.03 ± 0.002	−7.0 ± 0.14
Trp219	−2.7 ± 0.07	−1.3 ± 0.10	2.23 ± 0.08	−0.3 ± 0.005	−2.0 ± 0.06
Arg259	0.31 ± 0.125	−21.4 ± 0.49	14.3 ± 0.27	−0.13 ± 0.00	−7.0 ± 0.22

### 3.5 Covalent docking outcomes

**FIGURE 2:**
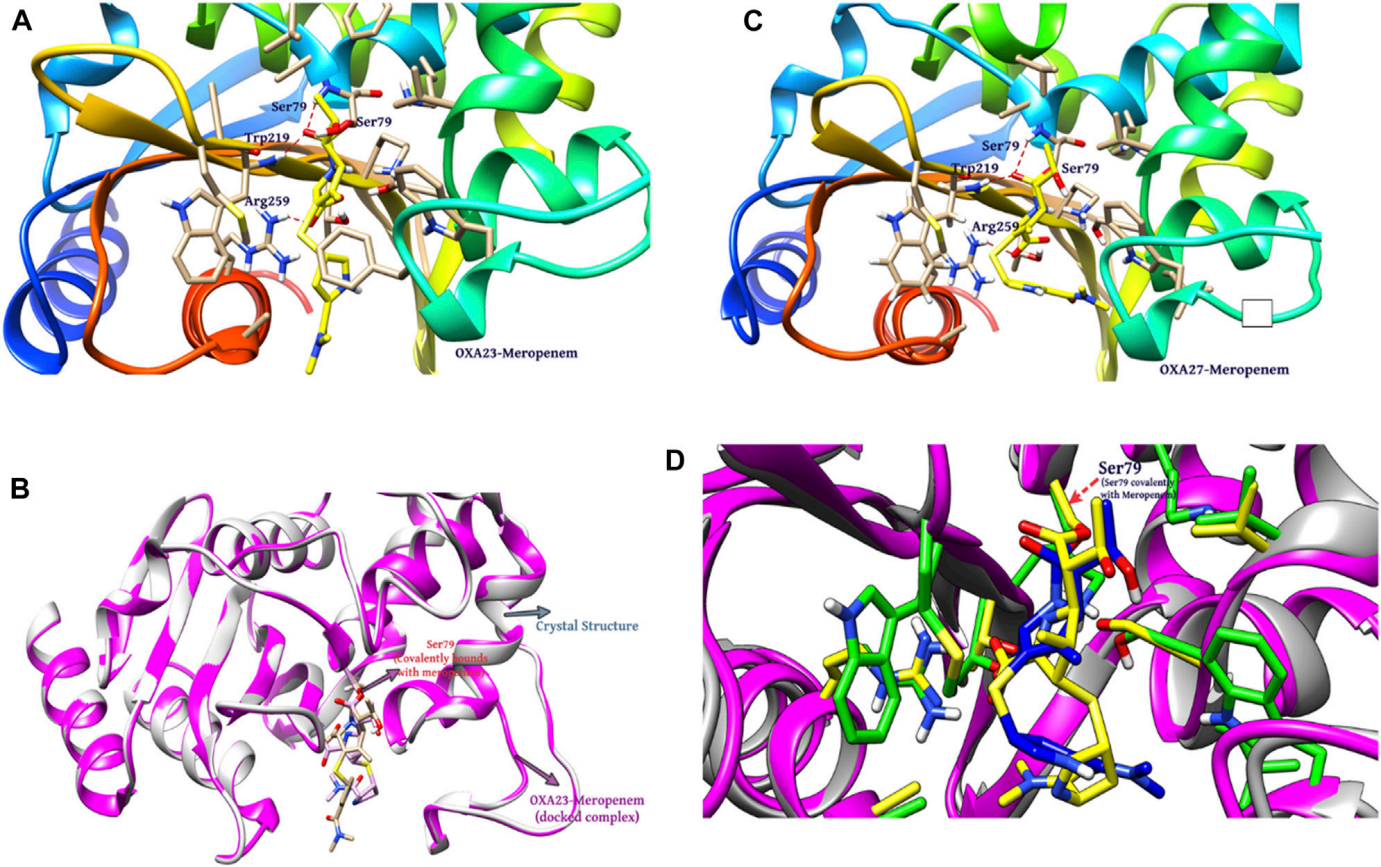
Interaction of meropenem with **(A)** OXA-23. **(B)** Superimposition of OXA-23 (docked complex, pink color) and the crystal structure (gray color) (results: 0.2 Å). **(C)** OXA-27. **(D)** Superimposition of OXA-23 (pink color) and OXA-27 (gray color) (results: 0.5 Å).

Covalent docking was performed from the outcome of the molecular docking analyses. The covalent docking analysis was performed to display how the transition state was supposed to be stabilized and the residues which contribute to the hydrogen bonding interactions. Furthermore, the transition state complex was stabilized by –NH of Ser79 and –NH of Trp219 (backbone), called the “oxyanion hole.” However, the outcome of meropenem, Pubchem_67943222, Pubchem_25224737, Pubchem_10645796, and ChEMBL_14 forms a “covalent bond” between O^−^of Ser79 and the carbonyl group of carbapenems. Meropenem has shown a CovDock energy value of −3.5 kcal mol^-1^ and -1.92 kcal mol^-1^ with OXA-23 and OXA-27, respectively. The analogs (Pubchem_67943222, Pubchem_25224737, Pubchem_10645796, and ChEMBL_14) showed CovDock energy ranging from −9.0 to −6.0 kcal mol^-1^ (Table 3). To identify the reliability of docking, the analyses were performed in triplicate. The average distance was measured between the protein and meropenem and their standard deviation values are given in Table 4. The covalent docking analysis evidenced that the complex was stabilized by the –NH group backbone of Trp219 and Ser79 with the carbonyl group. The hydrogen bonding interactions with the amino acid residues such as Ser126, Thr217, and Arg259 (Figure 2) are correlated with earlier studies (Smith et al., 2013).

**TABLE 3:** Covalent docking values of meropenem and its analogs against OXA-23 and OXA-27.

Compound	OXA-23	OXA-27
Meropenem	−3.5	−1.9
Pubchem_67943222	−9.0	−7.2
Pubchem_25224737	−8.2	−6.0
Pubchem_10645796	−8.2	−6.4
ChEMBL_14	−7.7	−6.0

**TABLE 4:** Top compounds from the outcome of covalent docking against OXA-23 and OXA-27.

a) OXA-23			
Sl. no.	Compound	Interactions	Mean distance (Å) and SD values are given in parenthesis
		Lys124 N—H … O	1.93 ± 0.045
		Leu125 N—H … O	1.92 ± 0.065
		Thr217 O–H … O	1.90 ± 0.081
		Arg259 N–H … O	1.93 ± 0.079
		Arg259 N—H … O	2.67 ± 0.205
1	Pubchem_10645796	Ser126 O–H … O	1.93 ± 0.125
		Ser79 N–H … O	2.27 ± 0.095
		Trp219 N–H … O	1.93 ± 0.149
		Leu125 O … H–O	1.89 ± 0.063
		Ser126 O–H … O	1.85 ± 0.054
		Thr217 O–H … O	1.96 ± 0.068
2	Pubchem_67943222	Arg259 N—H … O	1.96 ± 0.101
		Trp219 N—H … O	1.87 ± 0.107
		Ser79 N—H … O	2.25 ± 0.192
		Lys124 O–H … N	1.98 ± 0.098
		Leu125 O–H … N	2.06 ± 0.093
		Ser126 O–H … O	1.92 ± 0.075
		Thr217 O … H–O	1.94 ± 0.143
		Arg259 N—H … O	2.53 ± 0.105
		Arg259 N—H … O	1.88 ± 0.104
3	Pubchem_25224737	Trp219 O–H … O	2.17 ± 0.143
		Trp219 N—H … O	1.92 ± 0.106
		Ser79 N—H … O	2.13 ± 0.119
		Ser126 O–H … O	1.94 ± 0.128
		Thr217 O–H … O	1.90 ± 0.135
		Arg259 N—H … O	2.18 ± 0.110
		Arg259 N—H … O	2.02 ± 0.107
4	Meropenem	Trp219 N—H … O	2.08 ± 0.187
		Ser79 N—H … O	2.15 ± 0.099
6	ChEMBL_14	Ser79 N—H … O	2.17 ± 0.135
		Trp219 N—H … O	1.89 ± 0.153
		Ser126 O–H … O	1.97 ± 0.107
		Thr217 O–H … O	2.00 ± 0.200
		Arg259 N—H … O	2.57 ± 0.156
		Arg259 N—H … O	1.96 ± 0.152
		Trp219 O–H … O	2.12 ± 0.083

**TABLE 4:** (*Continued*) Top compounds from the outcome of covalent docking against OXA-23 and OXA-27.

b. OXA-27			
Sl. no.	Compound	Interactions	Mean distance (Å) and SD values are given in parenthesis
1	Pubchem_10645796	Lys124 N—H … O	1.94 ± 0.053
		Leu125 N—H … O	2.01 ± 0.700
		Thr217 O–H … O	1.93 ± 0.115
		Arg259 N–H … O	1.95 ± 0.090
		Arg259 N–H … O	2.65 ± 0.077
		Ser126 O–H … O	-
		Ser79 N–H … O	2.27 ± 0.10
		Trp219 N–H … O	1.89 ± 0.076
2	Pubchem_67943222	Leu125 O … H–O	1.97 ± 0.101
		Ser126 O–H … O	-
		Thr217 O–H … O	1.97 ± 0.090
		Arg259 N—H … O	2.05 ± 0.082
		Trp219 N—H … O	1.97 ± 0.150
		Ser79 N—H … O	2.43 ± 0.077
3	Pubchem_25224737	Lys124 O–H … N	1.93 ± 0.065
		Leu125 O–H … N	1.97 ± 0.110
		Ser126 O–H … O	2.09 ± 0.70
		Thr217 O … H–O	1.99 ± 0.127
		Arg259 N–H … O	1.98 ± 0.067
		Arg259 N–H … O	2.60 ± 0.127
		Trp219 O–H … O	-
		Trp219 N–H … O	1.87 ± 0.080
		Ser79 N–H … O	2.24 ± 0.098
6	Meropenem	Ser126 O–H … O	-
		Thr217 O–H … O	2.28 ± 0.0694
		Arg259 N–H … O	2.11 ± 0.114
		Arg259 N–H … O	2.23 ± 0.088
		Trp219 N–H … O	1.99 ± 0.098
		Ser79 N–H … O	2.43 ± 0.125
9	ChEMBL_14	Ser79 N—H … O	2.4 ± 0.104
		Trp219 N—H … O	1.87 ± 0.092
		Arg259 N—H … O	2.02 ± 0.140
		Arg259 N—H … O	2.55 ± 0.094
		Trp217 O–H … O	1.99 ± 0.148
	

The structural insight indicates that meropenem showed interactions with Ser126, Thr217, Arg259, Trp219, and Ser79 (catalytic sites). The compound Pubchem_10645796 formed interactions with Lys124, Leu125, Arg259, Ser79, and Trp219 in addition to the N-H....O interaction, where the -NH group in the backbone of Ser79 and –NH of Trp219 stabilizes the “oxyanion hole” which was evident in the molecular docking analysis. Furthermore, Thr217 and Ser126 showed stronger interactions of O–H...O with meropenem. We can also observe a similar hydrogen bonding interaction in Pubchem_67943222 but it lacks interaction with Leu125. Pubchem_25224737 has also shown O–H...O interactions with Lys124, Leu125 Ser126, Thr217, and Trp219 and Trp219, Ser79, and Arg259 of N-H...O interactions. ChEMBL_14 also showed interactions with Ser126, Thr217, Arg259, Trp219, and Ser79 similar to meropenem. From the outcome, OXA-27 produced similar interactions similar to OXA-23 with the meropenem analogs and the interaction with Ser126 is inconsistent. Hence, the compounds meropenem, Pubchem_25224737, Pubchem_10645796, and ChEMBL_14 are taken for further molecular dynamics simulation to understand the dynamic behaviour of the complexes.

### 3.6 Atomic-level large-scale analysis of conserved interactions

The nAPOLI webserver was employed to highlight the comprehensive reports of the interacting residues/atoms through interactive virtual representations. The study exhibits the residues Ala78, Phe110, Ser126, Val128, Leu166, Lys216, Thr217, Trp219, and Arg259 which are involved in the binding with meropenem and its analogs with various interaction types (Table 5). Furthermore, the analysis evidenced that acceptor, donor, and hydrophobic interactions could be useful to understand the atomic insights. These parameters confirmed the effective binding between the protein and ligand. In the present study, OXA-27 bound to meropenem and its analogs showing similar residual interactions to a crystal structure (OXA-23). Supplementary Table S2 summarizes the number of atoms involved in the interactions.

**TABLE 5:** nAPOLI analysis.

PDB Entry	Source residue name	Source residue number	Source chain	Interaction type
OXA-23_Meropenem	PHE	110	A	Hydrophobic
OXA-23_Meropenem	SER	126	A	Hydrogen bond
OXA-23_Meropenem	SER	126	A	Hydrogen bond (water)
OXA-23_Meropenem	VAL	128	A	Hydrophobic
OXA-23_Meropenem	TRP	165	A	Hydrophobic
OXA-23_Meropenem	THR	217	A	Hydrogen bond
OXA-23_Meropenem	TRP	219	A	Hydrogen bond
OXA-23_Meropenem	TRP	219	A	Hydrophobic
OXA-23_Meropenem	MET	221	A	Hydrophobic
OXA-23_Meropenem	ARG	259	A	Attractive
OXA-23_Meropenem	ARG	259	A	Hydrogen bond/attractive
OXA-27_Meropenem	ARG	259	A	Hydrogen bond/attractive
OXA-27_Meropenem	ARG	259	A	Hydrogen bond/attractive
OXA-27_Meropenem	ALA	78	A	Hydrophobic
OXA-27_Meropenem	PHE	110	A	Hydrophobic
OXA-27_Meropenem	PHE	110	A	Hydrophobic
OXA-27_Meropenem	SER	126	A	Hydrogen bond
OXA-27_Meropenem	VAL	128	A	Hydrophobic
OXA-27_Meropenem	LEU	166	A	Hydrophobic
OXA-27_Meropenem	LYS	216	A	Attractive
OXA-27_Meropenem	LYS	216	A	Repulsive
OXA-27_Meropenem	THR	217	A	Hydrogen bond
OXA-27_Meropenem	TRP	219	A	Hydrogen bond
OXA-27_Meropenem	TRP	219	A	Hydrophobic
OXA-27_Meropenem	ARG	259	A	Hydrogen bond/attractive
OXA-27_Meropenem	ARG	259	A	Hydrogen bond/attractive
OXA-27_Pubchem_10645796	SER	79	A	Hydrogen bond
OXA-27_Pubchem_10645796	PHE	110	A	Hydrophobic
OXA-27_Pubchem_10645796	PHE	110	A	Hydrophobic
OXA-27_Pubchem_10645796	PHE	110	A	Hydrophobic
OXA-27_Pubchem_10645796	PHE	110	A	Hydrophobic
OXA-27_Pubchem_10645796	PHE	110	A	Hydrophobic
OXA-27_Pubchem_10645796	PHE	110	A	Hydrophobic
OXA-27_Pubchem_10645796	PHE	110	A	Hydrophobic
OXA-27_Pubchem_10645796	TRP	113	A	Hydrophobic
OXA-27_Pubchem_10645796	VAL	128	A	Hydrophobic
OXA-27_Pubchem_10645796	LEU	166	A	Hydrophobic
OXA-27_Pubchem_10645796	LYS	216	A	Attractive
OXA-27_Pubchem_10645796	TRP	219	A	Hydrogen bond
OXA-27_Pubchem_10645796	ARG	259	A	Attractive
OXA-27_Pubchem_10645796	ARG	259	A	Hydrogen bond/attractive
OXA-27_Pubchem_25224737	SER	79	A	Hydrogen bond
OXA-27_Pubchem_25224737	PHE	110	A	Hydrophobic
OXA-27_Pubchem_25224737	PHE	110	A	Hydrophobic
OXA-27_Pubchem_25224737	PHE	110	A	Hydrophobic
OXA-27_Pubchem_25224737	TRP	113	A	Hydrophobic
OXA-27_Pubchem_25224737	SER	126	A	Hydrogen bond
OXA-27_Pubchem_25224737	VAL	128	A	Hydrophobic
OXA-27_Pubchem_25224737	LEU	166	A	Hydrophobic
OXA-27_Pubchem_25224737	LYS	216	A	Attractive
OXA-27_Pubchem_25224737	TRP	219	A	Hydrogen bond
OXA-27_Pubchem_25224737	ARG	259	A	Attractive
OXA-27_Pubchem_25224737	ARG	259	A	Hydrogen bond/attractive
OXA-27_Pubchem_67943222	SER	79	A	Hydrogen bond
OXA-27_Pubchem_67943222	PHE	110	A	Hydrophobic
OXA-27_Pubchem_67943222	PHE	110	A	Hydrophobic
OXA-27_Pubchem_67943222	PHE	110	A	Hydrophobic
OXA-27_Pubchem_67943222	PHE	110	A	Hydrophobic
OXA-27_Pubchem_67943222	PHE	110	A	Hydrophobic
OXA-27_Pubchem_67943222	TRP	113	A	Hydrogen bond
OXA-27_Pubchem_67943222	TRP	113	A	Hydrophobic
OXA-27_Pubchem_67943222	SER	126	A	Hydrogen bond
OXA-27_Pubchem_67943222	VAL	128	A	Hydrophobic
OXA-27_Pubchem_67943222	LEU	166	A	Hydrophobic
OXA-27_Pubchem_67943222	LYS	216	A	Attractive
OXA-27_Pubchem_67943222	THR	217	A	Hydrogen bond
OXA-27_Pubchem_67943222	TRP	219	A	Hydrogen bond
OXA-27_Pubchem_67943222	MET	221	A	Hydrophobic
OXA-27_Pubchem_67943222	ASP	222	A	Attractive
OXA-27_Pubchem_67943222	ASP	222	A	Hydrogen bond/attractive
OXA-27_Pubchem_67943222	ASP	222	A	Hydrogen bond/attractive
OXA-27_Pubchem_67943222	ASP	222	A	Attractive
OXA-27_Pubchem_67943222	ARG	259	A	Attractive
OXA-27_Pubchem_67943222	ARG	259	A	Hydrogen bond/attractive
OXA-27_ChEMBL_14	SER	79	A	Hydrogen bond
OXA-27_ChEMBL_14	PHE	110	A	Hydrophobic
OXA-27_ChEMBL_14	PHE	110	A	Hydrophobic
OXA-27_ChEMBL_14	PHE	110	A	Hydrophobic
OXA-27_ChEMBL_14	PHE	110	A	Hydrophobic
OXA-27_ChEMBL_14	PHE	110	A	Hydrophobic
OXA-27_ChEMBL_14	PHE	110	A	Hydrophobic
OXA-27_ChEMBL_14	PHE	110	A	Hydrophobic
OXA-27_ChEMBL_14	PHE	110	A	Hydrophobic
OXA-27_ChEMBL_14	TRP	113	A	Hydrophobic
OXA-27_ChEMBL_14	TRP	113	A	Hydrophobic
OXA-27_ChEMBL_14	SER	126	A	Hydrogen bond
OXA-27_ChEMBL_14	VAL	128	A	Hydrophobic
OXA-27_ChEMBL_14	LEU	166	A	Hydrophobic
OXA-27_ChEMBL_14	LYS	216	A	Attractive
OXA-27_ChEMBL_14	THR	217	A	Hydrogen bond
OXA-27_ChEMBL_14	TRP	219	A	Hydrophobic
OXA-27_ChEMBL_14	TRP	219	A	Hydrophobic
OXA-27_ChEMBL_14	TRP	219	A	Hydrophobic
OXA-27_ChEMBL_14	MET	221	A	Hydrophobic
OXA-27_ChEMBL_14	MET	221	A	Hydrophobic
OXA-27_ChEMBL_14	ARG	259	A	Hydrogen bond/attractive
OXA-27_ChEMBL_14	ARG	259	A	Hydrogen bond/attractive

### 3.7 Molecular dynamics simulations

The molecular dynamics simulation was performed to understand the conformational stability and flexibility of the structure. The system was subjected to 100 ns for the homology modeled structure and 50 ns for the protein–ligand complex. The detailed understanding of the stability on the simulated complexes was obtained from the RMSD plot. The molecular dynamics simulation was performed for the following complexes: OXA-27-meropenem, OXA-27-Pubchem_10645796, OXA-27-Pubchem_25224737, OXA-27-ChEMBL_14, and OXA-23-meropenem as a reference.

**FIGURE 3:**
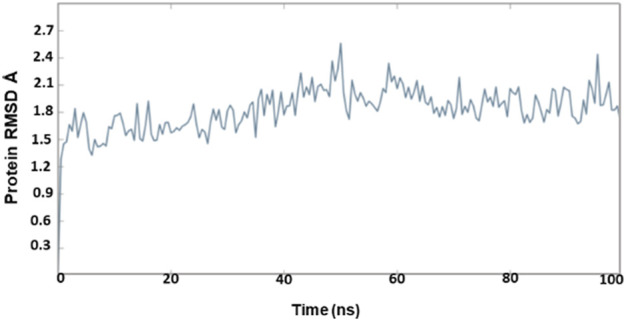
RMSD of OXA-27 from 100-ns molecular dynamics simulations.

**FIGURE 4:**
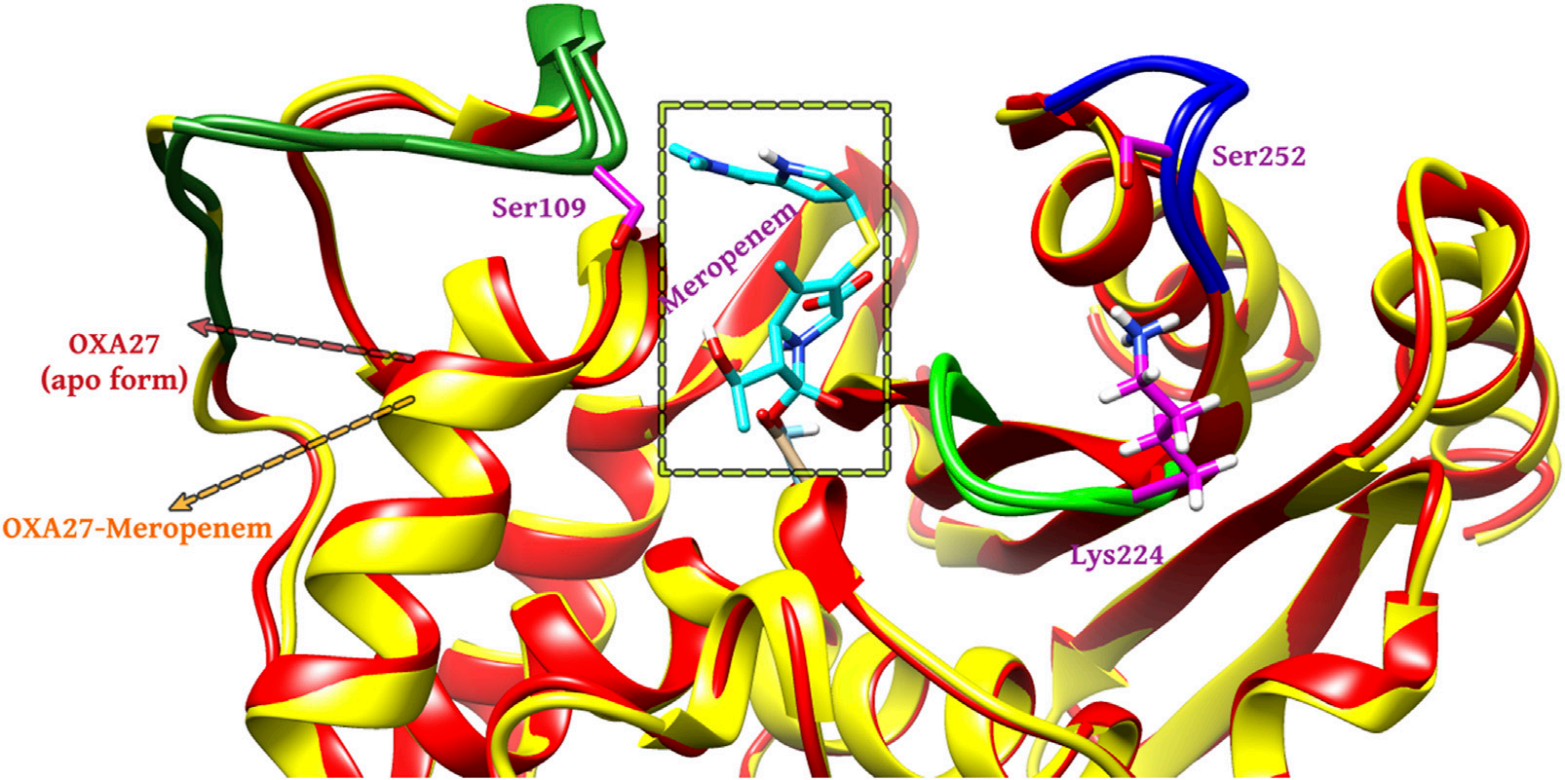
Molecular dynamics simulation analysis for the (A) *apo* form (red color) and (B) presence of meropenem (yellow color).

The outcome of the simulation reveals that the *apo* structure was equilibrated in a short time and that some fluctuations have occurred amid 1.5 and 2.4 Å between 40 and 60 ns. The results evidenced that the system was stable throughout the simulation (Figure 3). To understand further insights from the simulation outcomes related to the specific region of ligand binding, we observed the dynamic behaviour of the loop-containing regions during the simulation run. The residues Lys224—Ser252 from loop 1, Ser252—Ser109 from loop 2, and Ser109—Lys224 from loop 3 (residues taken for the measurement purpose) are shown in Figure 4. The distance of starting coordinates and initial, middle, and final frames of the molecular dynamics simulation are shown in Table 6. This study suggests that the protein tends to have a wide loop opening in the absence of meropenem. The distance between the loops was smaller in the presence of meropenem in the complex, *i.e.,* the loop regions are confined. Hence, the gap between the loops is very minimal in the presence of meropenem.

**TABLE 6:** Distance between starting coordinates and the final coordinates during the molecular dynamics run.

Sl. no.	Title	Description	Loop 1 (Lys224—Ser252)	Loop 2 (Ser252—Ser109)	Loop 3 (Ser 109—Lys 224)
1	Homology modeled structure OXA-27	Starting structure	12.70	15.23	19.68
		First frame	11.08	14.07	18.36
		Middle frame	10.25	18.87	22.17
		Last frame	10.96	17.23	22.11
2	Docked complex Pubchem_25224737	Starting structure	12.69	15.24	19.67
		First frame	10.88	14.51	19.59
		Middle frame	10.39	17.93	22.37
		Last frame	10.27	15.65	20.16

The OXA-23–meropenem complex has shown the peak ranging from 0.8 to 1.8 Å by producing a median-size gap between Cα and meropenem (Supplementary Figure S3). The molecular dynamics simulation reveals that Pubchem_10645796, Pubchem_25224737, and ChEMBL_14 are stable. Pubchem_10645796 was tightly bound to the protein although it has slight variation up to 30 ns and, later, it was stable throughout the simulation. Pubchem_25224737 was also constant throughout the simulation. The molecular dynamics simulation of the meropenem–OXA-27 complex has shown more fluctuations between 0.5 and 2.0 Å from 10 to 27 ns. Later, the complex was stable after 35 ns, and it was constant till the end of the simulation. In the case of ChEMBL_14, the RMSD was found to fluctuate throughout the simulation and increased up to 1.5 Å and maintains the close contact with the protein.

**FIGURE 5:**
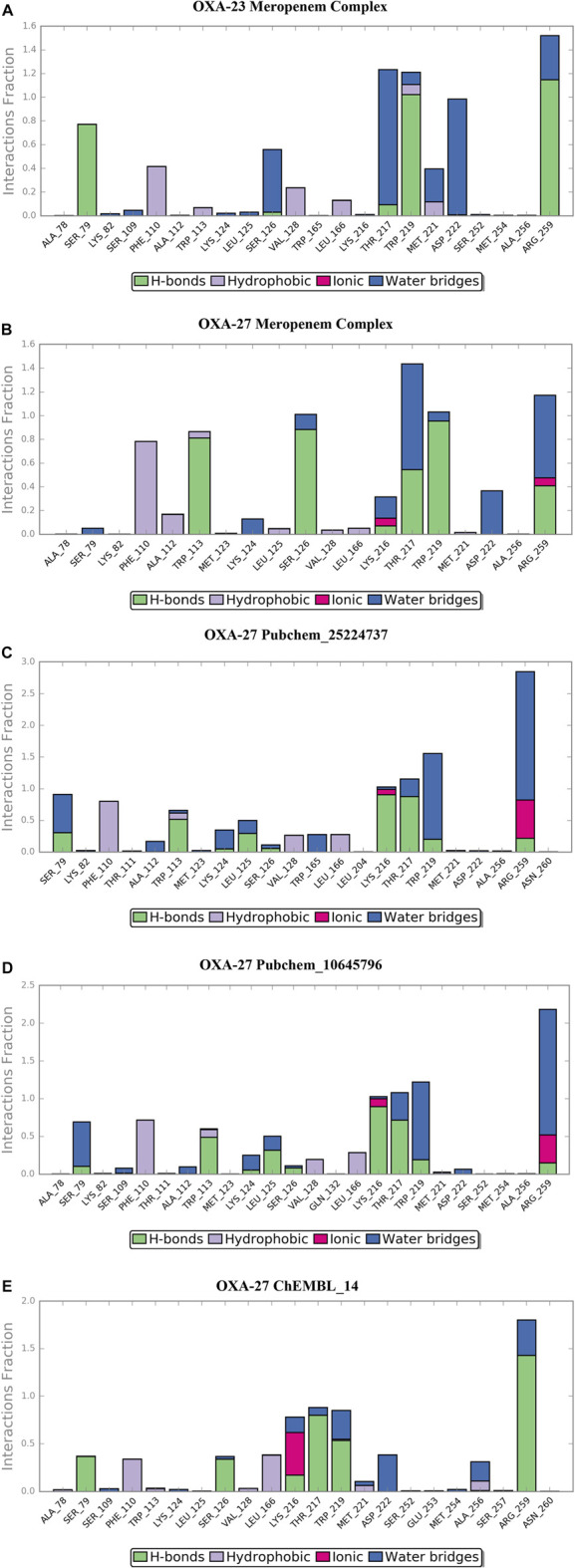
Desmond MD calculated protein–ligand contacts at the binding site of OXA-23 and OXA-27. **(A)** OXA-23–meropenem complex. **(B)** OXA-27–meropenem complex. **(C)** Pubchem_25224737. **(D)** Pubchem_10645796. **(E)** ChEMBL_14.

The location of the binding modes of compounds meropenem, Pubchem_10645796, Pubchem_25224737, and ChEMBL_14 was also confirmed by the complement of molecular dynamics simulation (Figure 5). Initially, we observed that the MDS of the OXA-23–meropenem complex showed major hydrogen bonding interactions with Ser79, Ser126, Trp219, Thr217, and Arg259 and it produced hydrophobic interactions with Phe110 Ala112 and minimal compactness with Trp113, whereas, Pubchem_25224737 showed significant hydrophobic interactions with Phe110, Trp113, Val128, and Leu166. The hydrogen bonding interactions are shown with Lys216, Thr217, Trp219, Arg259, and Asn260. Notably, the interactions with the water molecule improve the closeness of the complex. For instance, Lys216 plays a crucial role in hydrogen bonding, ionic interaction, and water bridges. Pubchem_10645796 showed interactions with Thr217, Trp219, and Arg259. It was stabilized by Arg259 and Lys216 through water, ionic, and hydrogen bonding interactions. ChEMBL_14 showed hydrophobic contacts with Phe110, Val128, and Leu166. It also showed interactions with Lys216, Thr217, Trp219, and Arg259 where these residues stabilize the complex through hydrogen bonding and water bridges; furthermore, the residues Lys216 and Arg259 showed ionic bond contacts. Trp113 showed its significance through hydrogen bonding and hydrophobic interactions with all the complexes. The RMSD value between the final frame of OXA-27–Pubchem_25224737 and the starting structure shows <1.0 Å and the frame-wise representation is given in Supplementary Figures S4, S5. Our docking analysis shows that Ser126 has inconsistent interactions. Harper et al. (2018) reported that the carboxylate region of the antibiotic interacts with the residues such as Arg259, Ser126, and Thr217 with the distance of 3.1 Å, 2.9 Å, and 2.5 Å, respectively. The molecular dynamics simulation confirms that Ser126 has shown interactions through the water molecule. The present study is in good agreement with earlier reports. Pubchem_67943222 does not show any significance in the molecular dynamics simulation.

In the presence of deuterium in Pubchem_25224737, the structure was closely observed to be related to the flexibility in the binding regions. The measured distance between the residues Ser109-Ser252, Ser252-Lys224, and Ser109-Lys224 in the loop were identified as 15.6 Å, 10.27 Å, and 20.16 Å, respectively. The RMSD value between the final frame of OXA-27–Pubchem_25224737 and the starting structure shows <1.0 Å and the frame-wise representation is given in Supplementary Figures S4, S5. Hence, in the presence of deuterium, conformational flexibility occurs which is in good agreement with the result reported by Huang et al. (2021).

### 3.8 DFT studies

To evaluate the orbital energy-level behaviour of the compounds, the HOMO and LUMO analyses were performed (Ramachandran, et al., 2022a). The difference between the orbital energy is given in Table 7. The gap energy values were in the range between 0.02 and 0.23 eV. A lower energy gap indicates higher kinetic energy and high chemical reactivity (Miar et al., 2021). Meropenem and its analogs have better energy values in the gap intervals. Notably, Pubchem_10645796 and ChEMBL_14 showed significant values when compared to meropenem. Hence, the reported compounds are in good agreement with our earlier studies. The lowest HOMO–LUMO gap in the aforementioned compounds showed that HOMO of the compound may transfer its electrons to less energy, LUMO, and it may have increased binding affinity with the protein to enhance their activities.

**TABLE 7:** Calculation of HOMO–LUMO values by B3LYP/6-31+G* methods.

a	Compound	HOMO	LUMO	ΔE (eV)
		Energy (eV)	Energy (eV)	
1	Meropenem	−0.22674	−0.01308	0.21366
2	Pubchem_10645796	−0.23518	−0.09011	0.14507
3	Pubchem_25224737	−0.22783	−0.04537	0.18246
4	Pubchem_2922432	−0.22778	−0.04536	0.18242
5	ChEMBL_14	−0.22723	−0.07117	0.15606

### 3.9 Spectrophotometric analysis

**FIGURE 6:**
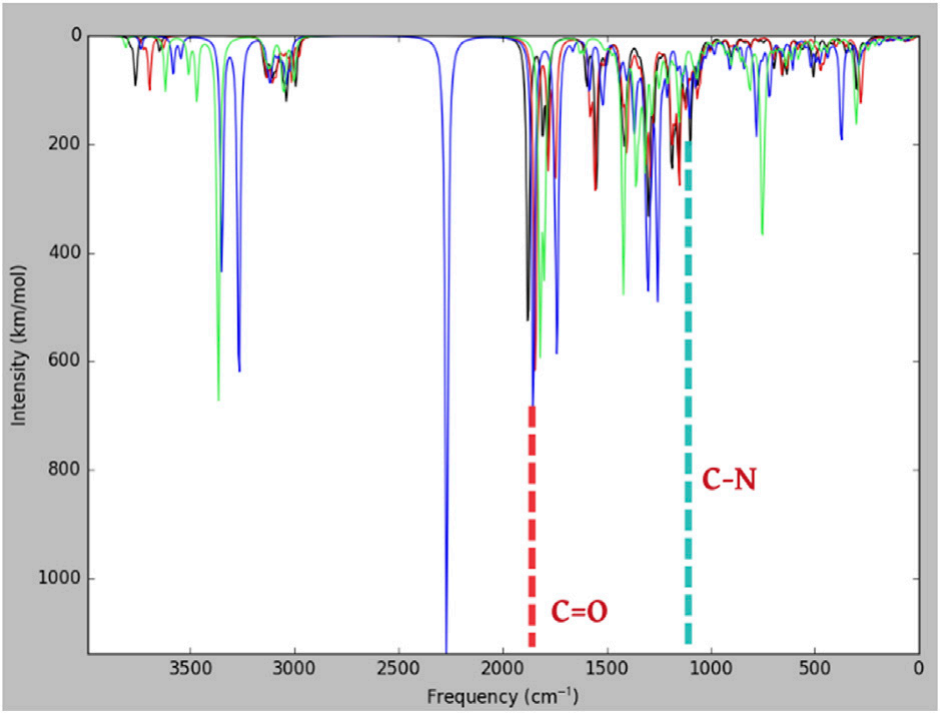
Predicted IR spectra (B3LYP/6-31+G^*^) for meropenem and its analogs.

Madhavi Stastry et al. (2013) reported the frequency of meropenem obtained through experimental and theoretical values (B3LYP/6-31+G^*^). In the present study, many bands corresponding to the vibration of beta-lactams were identified in the IR absorption of meropenem and its analogs (Figure 6). The bands show components corresponding to the stretching vibration of the C-N bond in the beta-lactam ring at 1,135 cm^−1^. The stretching of C=O bonds and C–C bonds is observed between 1,600 and 1,900 cm^−1^. Hence, our study evidenced that the stretching vibration of the C=O bond in the β-lactam ring was located at 1,843 cm^−1^ for meropenem, 1,853 cm^−1^ for Pubchem_25224737, 1,877 cm^−1^ for ChEMBL_14, and 1,819 cm^−1^ for Pubchem_10645796. The C-N bond also showed a frequency of 1,125 cm^−1^, 1,128 cm^−1^, 1,155 cm^−1^, and 1,087 cm^−1^, respectively. This is in concurrence with the experimental values of 1,887 and 1,135 cm^-1^ reported by Piontek et al. (2013). The IR spectrum of meropenem analogs shows the bands due to the stretching vibration of the ion pair complex C=O and C-N bonds, thus confirming the formation of the ion pair complex. Hence, C=O has the capacity to form the “oxyanion hole” with Trp219 and Ser79 (backbone -NH) after the nucleophilic attack. Then, C-N will have the ability to cleave the bond after hydrolysis. The predicted band values of the analogs lie in the experimental value range.

## 4 Discussion

The outcomes of the molecular docking analysis were compared with the experimental values, *viz.,* kinetic values and MIC values against OXA-23 and OXA-27. The covalent docking energy values and hydrogen bonding interactions are the parameters we considered to determine the carbapenemase activity.

Evans and Amyes (2014) reported that the K_m_ values of meropenem against OXA-23 and OXA-27 were <1 and 15 uM, respectively. Furthermore, Sinem (2012) showed that the K_m_ value of meropenem against OXA-23 (maltose-binding protein) was about 17 (µM). In the same report, the K_cat_ value of the meropenem–OXA-23 complex was 0.068 s^−1^ which equals an activation free energy of ∼19 kcal mol^−1^. Rosenthal et al. (2010) reported that the experimental pI value ranges between 6.65 (OXA-23) and 6.8 (OXA-27) and the theoretical pI of OXA-23 and OXA-27 were 7.7 and 8.37, respectively. They clearly elaborated that the increase in the pI value of OXA-27 is due to substitution at the 247th position (Asn → Lys) where the pI value of Asn is 5.47 and Lys is 9.74. Furthermore, the kinetic values of meropenem against Singapore I-16 isolate possessing OXA-27 have shown a K_m_ value of 15 µM. A lower K_m_ corresponds to a higher binding affinity (Mariya and David et al., 2001). The steady state kinetics explores their significance on developing potent covalent inhibitors to improvise the activation of the enzyme. Furthermore, the MIC values play a vital role, which highlights the potency of an antibiotic to kill a pathogen. The present CovDock analysis of meropenem and its analogs showed about six hydrogen bonding interactions against OXA-23. The MIC value of meropenem in the A2265 strain showed about 32 μg mL^−1^ against A2265, whereas it registers 1 μg mL^−1^ against the ΔA2265 strain + OXA-23 (Yang et al., 2019). Furthermore, Smith et al. (2013) stated that the MIC value increased up to 128 times for meropenem against the ATCC17978 strain, and as a result, it exhibits resistance with meropenem. Our analysis showed a CovDock energy between −3.5 and −1.92 kcal mol^-1^ for meropenem against OXA-23 and OXA-27, respectively. On the other hand, the CovDock energy values for the compounds Pubchem_25224737, Pubchem_10645796, and ChEMBL_14 range between −9.0 and −6.0 kcal mol^-1^. Importantly, the Ser126, Thr217, Trp219, and Arg259 residue plays a crucial role in stabilizing the complex which is consistent to the results of the binding free energy analysis. Smith et al. (2013) elaborated that lower MIC values are related to bactericidal activity. The present study suggests that correlating the CovDdock energy values and hydrogen bonding interactions along with the MIC values could serve as a better tool to suggest the amount required of MIC to show their bactericidal activity. Therefore, OXA-27 showed better interactions with the highest hydrolytic activity against ChEMBL14 followed by Pubchem_252224737 and Pubchem_10645796 with the obtained results in line with the MIC values.

Our result indicates that the conformational dynamics of the interdomain interaction could be important to improve inhibitory binding, suggesting that the relative orientation of the identified domains of OXA-27 could be impacted by the flexibility of the interdomain linker. Such interdomain dynamics allowed these identified compounds to adapt onto a concave interface to bind to a variety of β-lactamases.

## 5 Concluding remarks

Treating infections caused by OXA-23 produced by *A. baumannii* has become a major concern in clinical settings. We attempted to identify the best lead compounds against OXA-23-producing *A. baumannii* using molecular docking, dynamics studies, and DFT approaches. Based on the obtained outcomes, the compounds Pubchem_25224737, Pubchem_10645796, and ChEMBL_14 are identified as the appropriate meropenem analogs which could enhance antibacterial activity to overcome microbial resistance. The spectrophotometric analysis also showed evidence of C=O and C-N atoms by showing the bands of 1,800 and 1,125 cm^-1^, respectively, and these values are compared with the experimental value range. This study indicates that increased binding affinity between the complexes resulted in the enhancement of the rate of acylation. However, there are no earlier studies on the OXA-23 variants. Our results highlight the importance of OXA-23 molecular docking studies and their compliance with the phenotypic results. Overall insights into the mechanism underlying the broad and enhanced binding of identified compounds toward OXA-23 and OXA-27 are presented in this study. These findings revealed the role of the inhibitor protein from the perspective of protein dynamics. The identified compounds with enhanced potency exhibited critical changes in the plasticity of interfacial loops and interdomain orientation. Such insights into the conformational dynamics can provide general guidelines toward the designing of novel inhibitors. The present study explored the potential compounds against OXA-23-producing *A. baumannii* and these outcomes will help in designing novel drugs to combat the problem of multidrug resistance in *A. baumannii*.

## Data Availability

The datasets presented in this study can be found in online repositories. The names of the repository/repositories and accession number(s) can be found in the article/Supplementary Material.
