# Supplementary material for: Computational exploration of molecular flexibility and interaction of meropenem analogs with the active site of oxacillinase-23 in Acinetobacter baumannii
Source: Front Chem. 2023 Feb 23;11:1090630. doi: 10.3389/fchem.2023.1090630 (PMC9996302; doi:10.3389/fchem.2023.1090630)
Supplement: Supplementary file 1 [file DataSheet2.docx]

**Computational Exploration of Molecular Flexibility and Interaction of meropenem analogues with active site of Oxacillinase-23 in *Acinetobacer baumannii.***

**Balajee Ramachandran^1^, Saravanan Muthupandian^2^, Jeyakanthan Jeyaraman^1*^, Bruno Silvester Lopes ^4,5*^**

^1^Structural Biology and Bio-Computing Lab, Department of Bioinformatics, Alagappa University, Karaikudi 630 004, Tamil Nadu, India

^2^Department of Pharmacology, Saveetha Institute of Medical and Technical Sciences (SIMATS), Chennai 600 077, Tamil Nadu, India

^3^School of Health and Life Sciences, Teesside University, Middlesbrough TS1 3BA, UK

^4^School of Health and Life Sciences, Teesside University, Middlesbrough TS1 3BA, UK

^5^National Horizons Centre, Teesside University, Darlington DL1 1HG, UK

*** Correspondence:**

**1. Dr. Jeyaraman Jeyakanthan**

**Professor. & Head**

Emails: jjkanthan@gmail.com, jjeyakanthan@alagappauniversity.ac.in

**2. Dr. Bruno Silvester Lopes**

Email: b.lopes@tees.ac.uk, brunoldlopez@gmail.com

**Supplementary Table**

**Fig. S1 Chemical compounds of the meropenem analogs with the SMILE Notation**

| **Sl. No.** | **SMILE NOTATION** | **CHEMICAL NAME** |
| --- | --- | --- |
| Meropenem | CC1C2C(C(=O)N2C(=C1SC3CC(NC3)C(=O)N(C)C)C(=O)O)C(C)O | 5-(dimethylcarbamoyl)pyrrolidin-3-yl]sulfanyl-6-[(1*R*)-1-hydroxyethyl]-4-methyl-7-oxo-1-azabicyclo[3.2.0]hept-2-ene-2-carboxylic acid |
| Pubchem_25224737 | CC1C2C(C(=O)N2C(=C1SC1CC(NC1)CNS(=O)(=O)N)C(=O)O)C(C)O | 6-(1-hydroxyethyl)-4-methyl-7-oxo-3-((5-((sulfamoylamino)methyl)pyrrolidin-3-yl)thio)-1-azabicyclo[3.2.0]hept-2-ene-2-carboxylic acid |
| Pubchem_67943222 | CC(C1C2C(C(=C(N2C1=O)C(=O)O)SCCO)CCCNCN)O | 4-(3-((aminomethyl)amino)propyl)-6-(1-hydroxyethyl)-3-((2-hydroxyethyl)thio)-7-oxo-1-azabicyclo[3.2.0]hept-2-ene-2-carboxylic acid |
| Pubchem_10645796 | CC1C2C(C(=O)N2C(=C1SC3CC(NC3)CNS(=O)(=O)N)C(=O)O)C(C)O | (4R,5beta)-3-[[(3R)-5beta-(Aminosulfonylaminomethyl)pyrrolidin-3alpha-yl]thio]-4alpha-methyl-6beta-[(R)-1-hydroxyethyl]-7-oxo-1-azabicyclo[3.2.0]hept-2-ene-2-carboxylic acid |
| ChEMBL_14 | CC1C2C(C(=O)N2C(=C1SC1CC(OC1)C(N)C(O)C)C(=O)O)C(C)O | 3-((2-(acetamidomethyl) tetrahydrofuran-3-yl)thio)-6-(1-hydroxyethyl)-4-methyl-7-oxo-1-azabicyclo[3.2.0]hept-2-ene-2-carboxylic acid |

**Table S2. Data Summary of meropenem and their analogues against OXA23 and OXA27.**

| Sl. No. | PDB | Ligand | Acceptor Atm | Aromatic Atm | Donor Atm | Hydrophobic Atm | Negative Atm | Positive Atm | Unrated Atm | Num Atoms | Aromatic Stacking | Hydrogen Bond | Hydrogen Bond Water | Hydrophobic Interaction | Repulsive Interaction | Attractive Interaction | Num Interactions |
| --- | --- | --- | --- | --- | --- | --- | --- | --- | --- | --- | --- | --- | --- | --- | --- | --- | --- |
| 1 | **OXA_23** | **Meropenem** | **6** | **0** | **4** | **7** | **2** | **2** | **11** | **26** | **0** | **7** | **2** | **19** | **0** | **5** | **13** |
|  |  |  |  |  |  |  |  |  |  |  |  |  |  |  |  |  |  |
| 2 | **OXA_27** | **Meropenem** | **8** | **0** | **5** | **7** | **2** | **2** | **11** | **26** | **0** | **5** | **0** | **6** | **1** | **3** | **13** |
| 3 | **OXA_27** | **Pubchem_10645796** | **10** | **0** | **6** | **7** | **3** | **1** | **9** | **27** | **0** | **3** | **0** | **10** | **0** | **3** | **15** |
| 4 | **OXA_27** | **Pubchem_25224737** | **10** | **0** | **6** | **7** | **3** | **1** | **9** | **27** | **0** | **4** | **0** | **6** | **0** | **3** | **12** |
| 5 | **OXA_27** | **Pubchem_67943222** | **9** | **0** | **6** | **7** | **3** | **2** | **8** | **24** | **0** | **8** | **0** | **9** | **0** | **7** | **21** |
| 6 | **OXA_27** | **ChEMBL_14** | **8** | **0** | **4** | **8** | **2** | **0** | **9** | **26** | **0** | **5** | **0** | **17** | **0** | **3** | **23** |
