# Supplementary material for: Computational exploration of molecular flexibility and interaction of meropenem analogs with the active site of oxacillinase-23 in Acinetobacter baumannii
Source: Front Chem. 2023 Feb 23;11:1090630. doi: 10.3389/fchem.2023.1090630 (PMC9996302; doi:10.3389/fchem.2023.1090630)
Supplement: Supplementary file 2 [file DataSheet1.docx]

**Computational Exploration of Molecular Flexibility and Interaction of meropenem analogues with active site of Oxacillinase-23 in *Acinetobacer baumannii.***

**Balajee Ramachandran^1^, Saravanan Muthupandian^2^, Jeyakanthan Jeyaraman^1*^, Bruno Silvester Lopes ^4,5*^**

^1^Structural Biology and Bio-Computing Lab, Department of Bioinformatics, Alagappa University, Karaikudi 630 004, Tamil Nadu, India

^2^Department of Pharmacology, Saveetha Institute of Medical and Technical Sciences (SIMATS), Chennai, Tamil Nadu, India

^3^School of Health and Life Sciences, Teesside University, Middlesbrough TS1 3BA, UK

^4^School of Health and Life Sciences, Teesside University, Middlesbrough TS1 3BA, UK

^5^National Horizons Centre, Teesside University, Darlington DL1 1HG, UK

*** Correspondence:**

**1. Dr. Jeyaraman Jeyakanthan**

**Professor. & Head**

Emails: jjkanthan@gmail.com, jjeyakanthan@alagappauniversity.ac.in

**2. Dr. Bruno Silvester Lopes**

Email: b.lopes@tees.ac.uk, brunoldlopez@gmail.com

**Supplementary Figures**

**Figure S1. Sequence alignment of OXA variants with OXA-23 using ClustalW and the figures was produced using Espript (Robert & Gouet, 2014)**

**A**

**
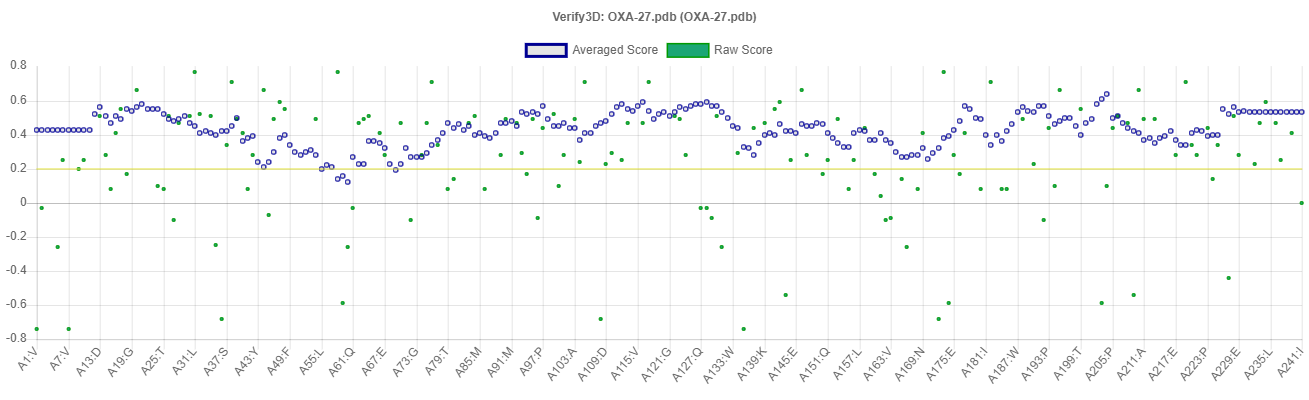
**

**B**

**C**

**C**

**Figure. S2 A) Structure validation with ERRAT tool**. The result of ERRAT tool shows that overall quality factor of the modelled protein based on various sorts of atoms were found to be 91.2 which are satisfactory. **B) Analysis of 3D-1D score of modelled protein by Verify 3D**. The figure shows that 74.56% of the residues had an average of 3D-1D score >=0.2 that is acceptable for our modelled protein. **C)** Ramachandran Plot Analysis showing that 93.9% are in most favoured regions and 5% are in allowed regions. **D)** **The OXA-23 crystal structure has been superimposed with their variants** [OXA-27, OXA-49, OXA-73, OXA-103, OXA-133, OXA-134, OXA-146, OXA-165, OXA-171, OXA-225]. The RMSD value is 0.3Å**. E**) Region RMSD alignment between OXA23 and OXA27, the 95^th^ position A – T and 247^th^ position K – N has shown 0.17Å and 0.13Å respectively.


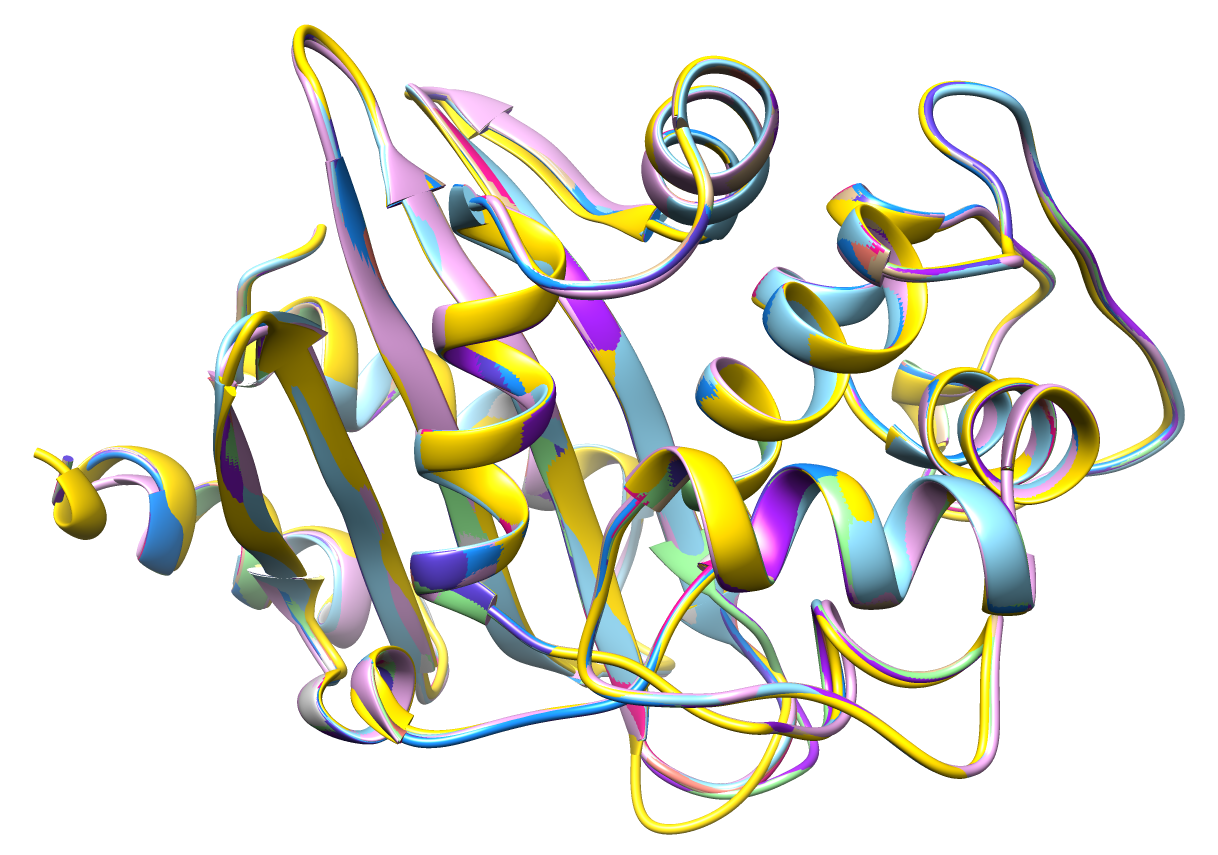


**D**


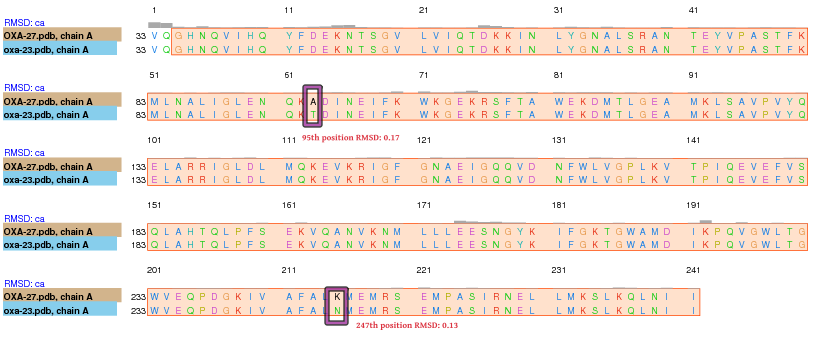


**E**

**Figure. S2 A) Structure validation with ERRAT tool**. The result of ERRAT tool shows that overall quality factor of the modelled protein based on various sorts of atoms were found to be 91.2 which are satisfactory. **B) Analysis of 3D-1D score of modelled protein by Verify 3D**. The figure shows that 74.56% of the residues had an average of 3D-1D score >=0.2 that is acceptable for our modelled protein. **C)** Ramachandran Plot Analysis showing that 93.9% are in most favoured regions and 5% are in allowed regions. **D)** **The OXA-23 crystal structure has been superimposed with their variants** [OXA-27, OXA-49, OXA-73, OXA-103, OXA-133, OXA-134, OXA-146, OXA-165, OXA-171, OXA-225]. The RMSD value is 0.3Å**. E**) Region RMSD alignment between OXA23 and OXA27, the 95^th^ position A – T and 247^th^ position K – N has shown 0.17Å and 0.13Å respectively.

**
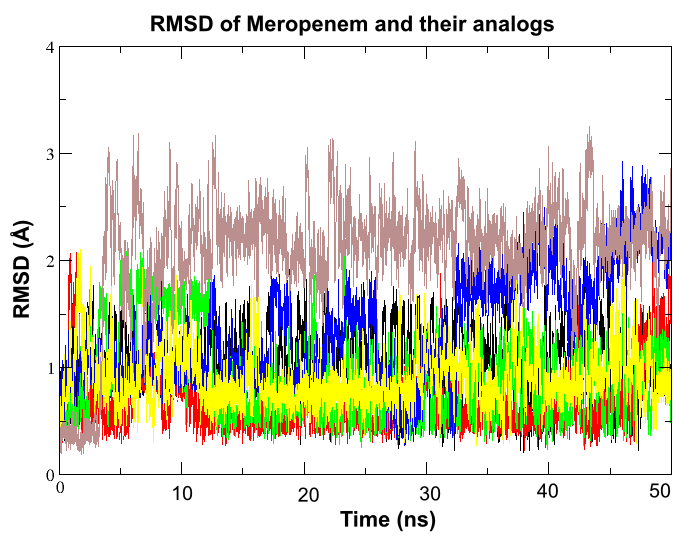
Figure S3** The RMSD analysis of meropenem (OXA23), Black; meropenem (OXA27), Blue; Pubchem_67943222 (OXA27), Brown; ChEMBL_14 (OXA27), Green; Pubchem_25224737 (OXA27), Red; Pubchem_10645796, Yellow.

**
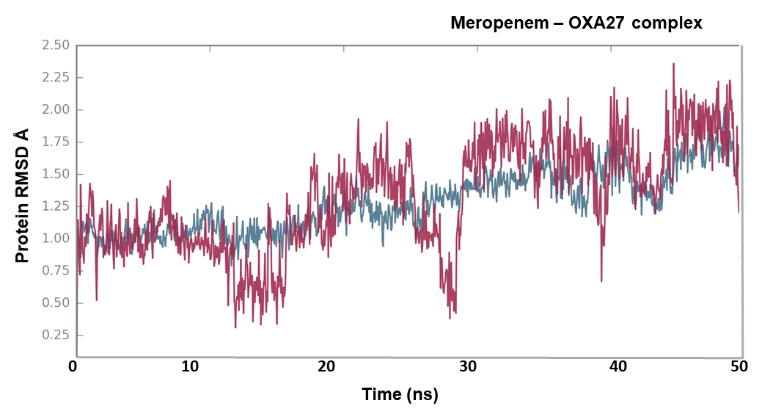

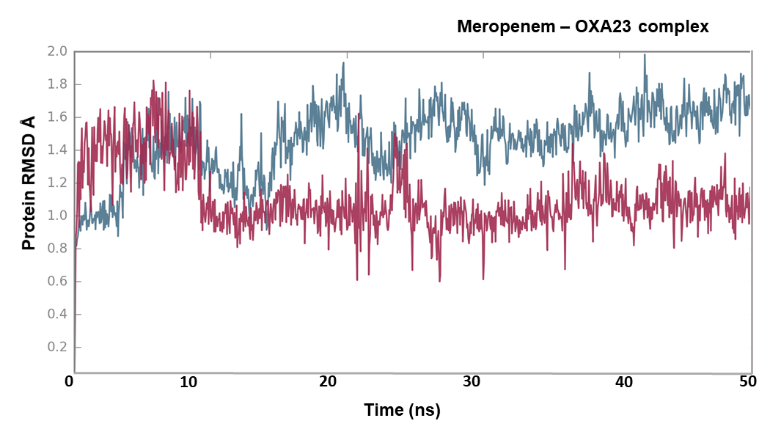
**

**B**

**A**


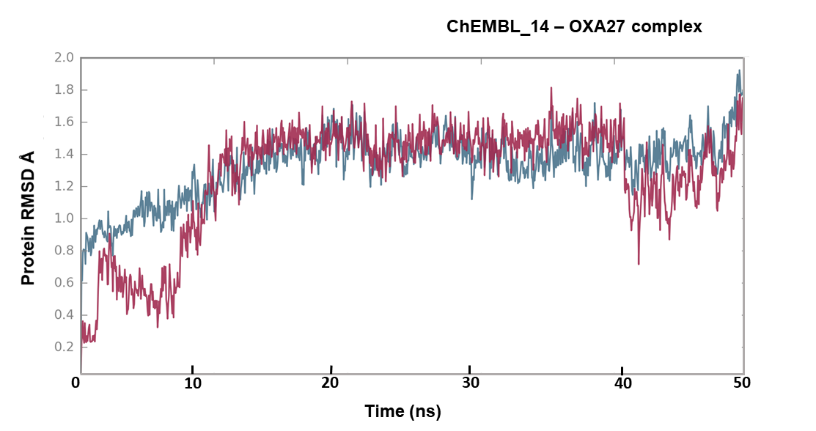
**
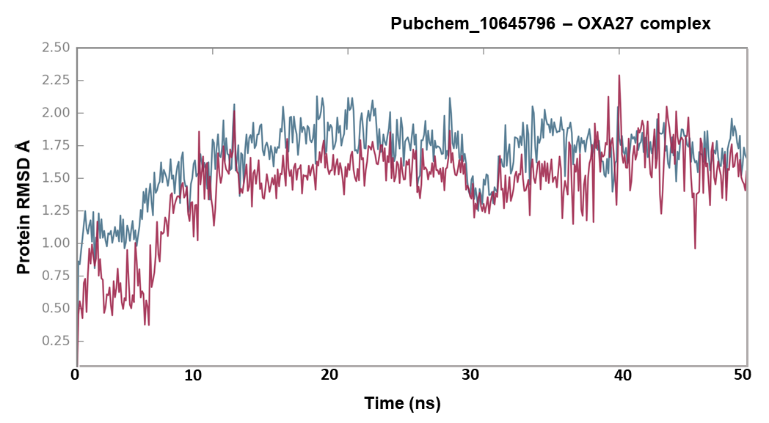

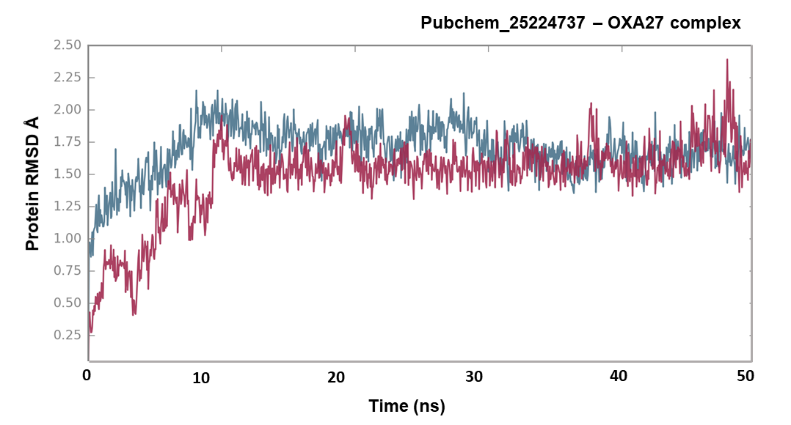
**

**E**

**D**

**C**

**Figure S4.** The molecular dynamics simulation of the following compounds. A) OXA-23 Meropenem complex B) Meropenem-OXA27 complex C) Pubchem_25224737- OXA27 complex D) Pubchem_10645796- OXA27 complex E) ChEMBL_14- OXA27 complex**.**


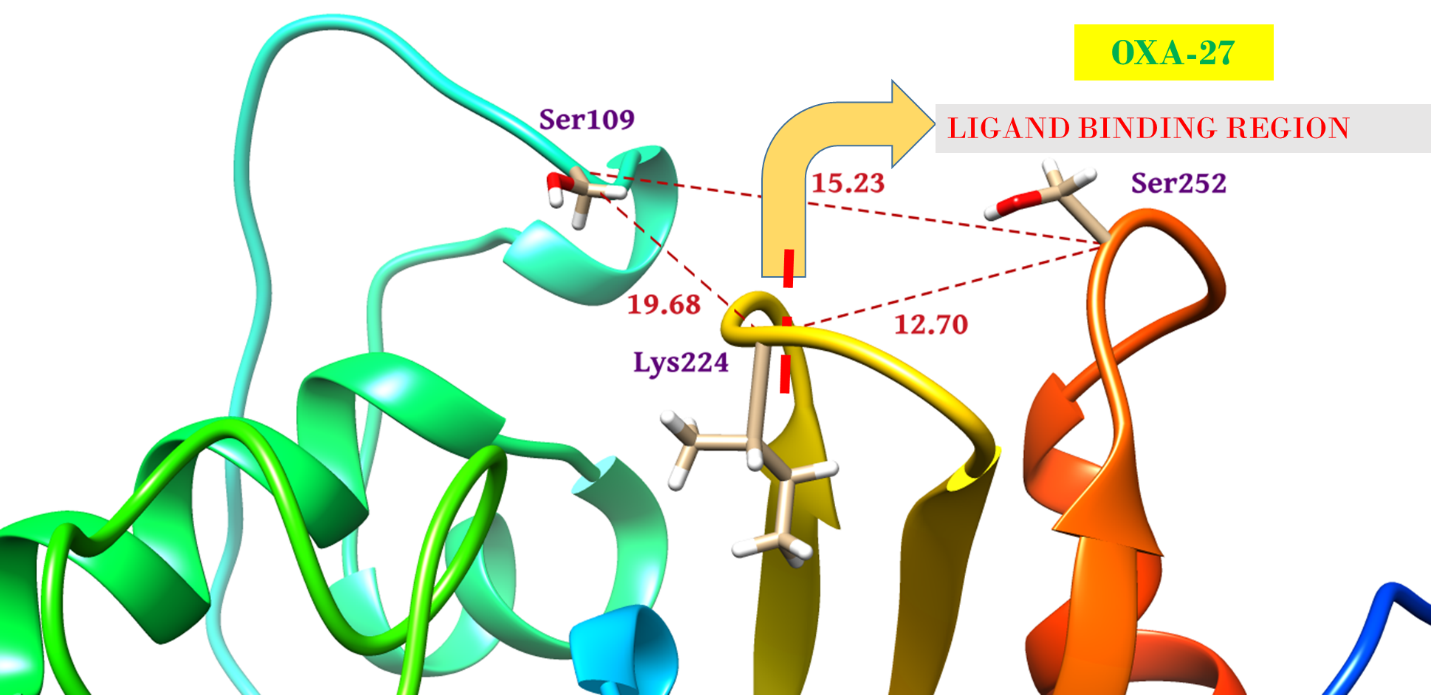


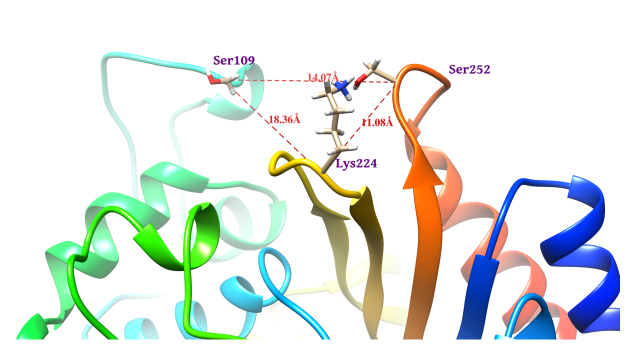


**Initial Frame**


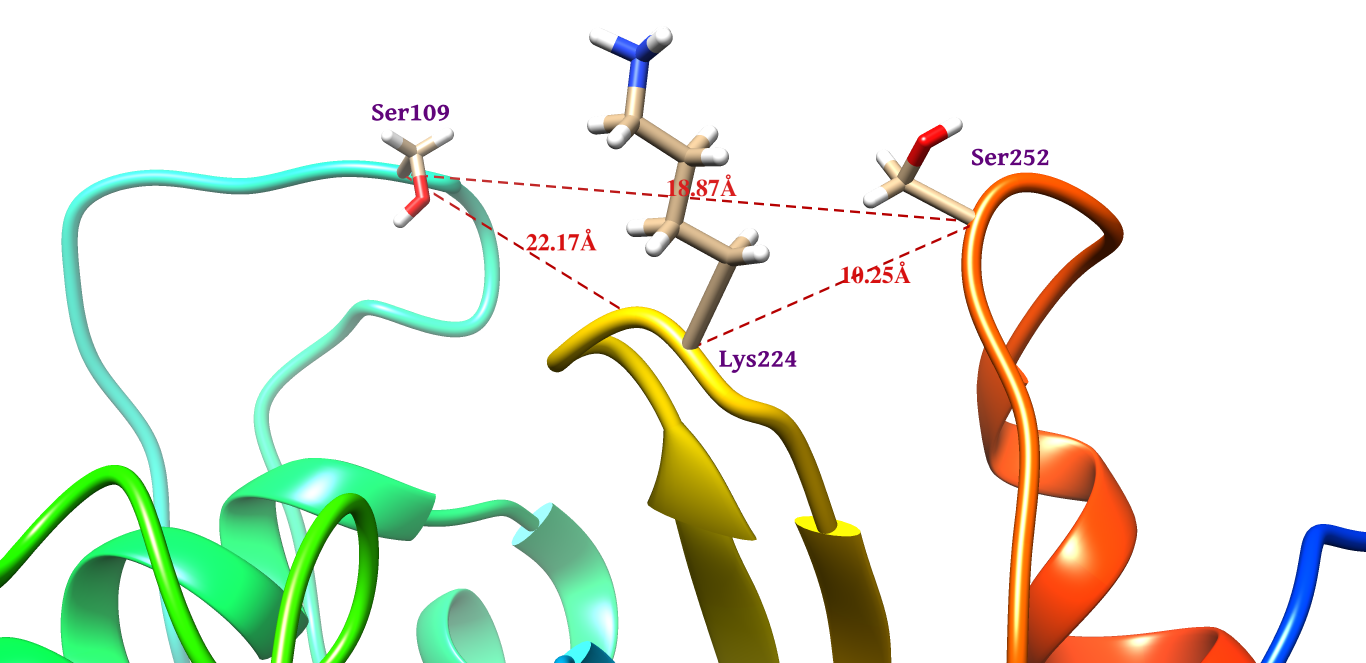


**Middle Frame**


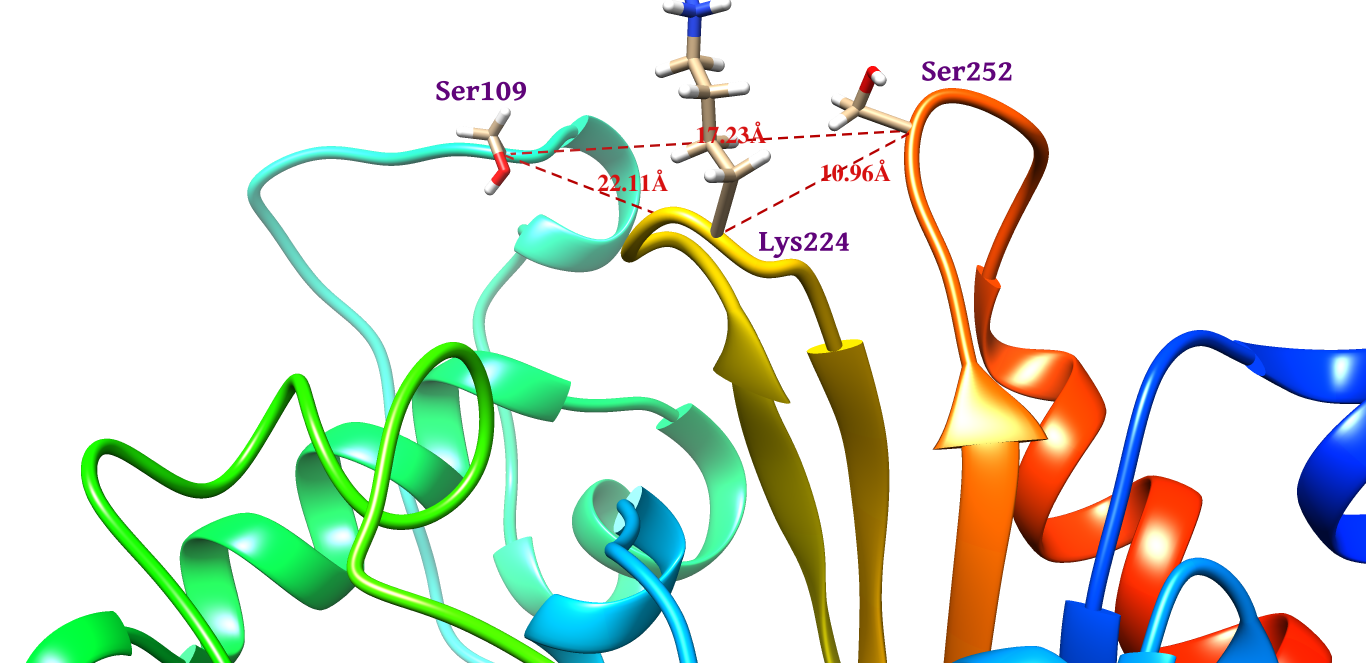


**Final Frame**

**Figure S5** Frame wise representation of apo form of OXA-27 in the molecular dynamics simulation represents loop distance evolution throughout the time. **A.** The figure depicts of ligand positioning in that loop and distance between C-α of residues Ser109, Lys224 and Ser252 to observe the loop behavior in the absence of the ligand (compared with initial, middle and final frames). The ligand enters the loop through this loop which was observed in the molecular dynamics simulation. The distance of the loop region was measured. The C-α of residues Ser109, Lys224 and Ser252 were taken for measurement purpose.

**
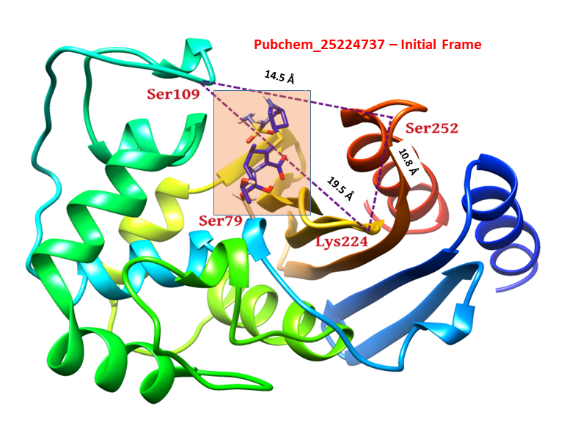
**

**
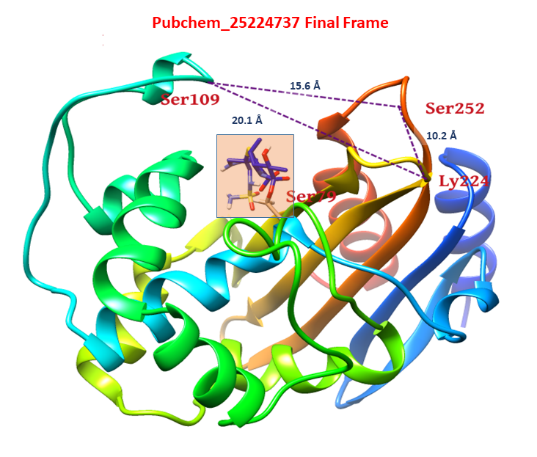

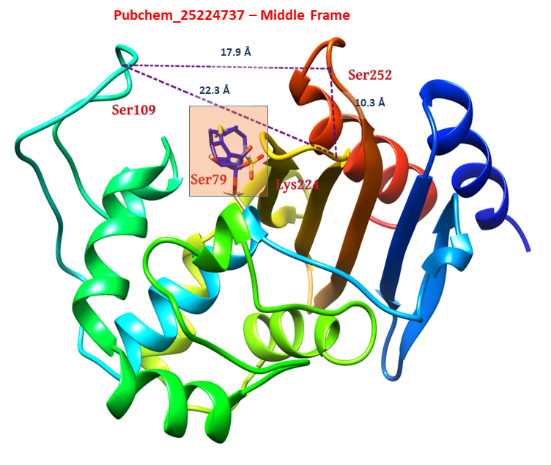
**

**Figure S6** Frame wise representation of meropenem analogue Pubchem_25224737 bounds with OXA-27 represents the loop distance evolution throughout the time. The distance of the loop region was measured. The Cα of residues Ser109, Lys224 and Ser252 were taken for measurement purpose.
